# Supplementary material for: Estimation of spatial demographic maps from polymorphism data using a neural network
Source: Mol Ecol Resour. Author manuscript; Available in PMC 2025 Jul 20. (PMC12276964; doi:10.1111/1755-0998.14005)

MOLECULAR ECOLOGY RESOURCES

Supplemental Information for:

Estimation of spatial demographic maps from polymorphism data using a neural network

Chris C. R. Smith, Gilia Patterson, Peter L. Ralph, and Andrew D. Kern

Table of Contents:

**Figure S1**	Page 2
**Figure S2**	Page 3
**Figure S3**	Page 5
**Figure S4**	Page 6
**Figure S5**	Page 7
**Figure S6**	Page 7
**Figure S7**	Page 8
**Figure S8**	Page 8
**Figure S9**	Page 9
**Figure S10**	Page 10
**Figure S11**	Page 11
**Figure S12**	Page 12
**Figure S13**	Page 13
**Figure S14**	Page 14
**Figure S15**	Page 15

MOLECULAR ECOLOGY
RESOURCES

Figure S1. PNG renderings for a random selection of training maps for the benchmark analysis. The blue channel conveys dispersal rate and the red channel conveys carrying capacity.

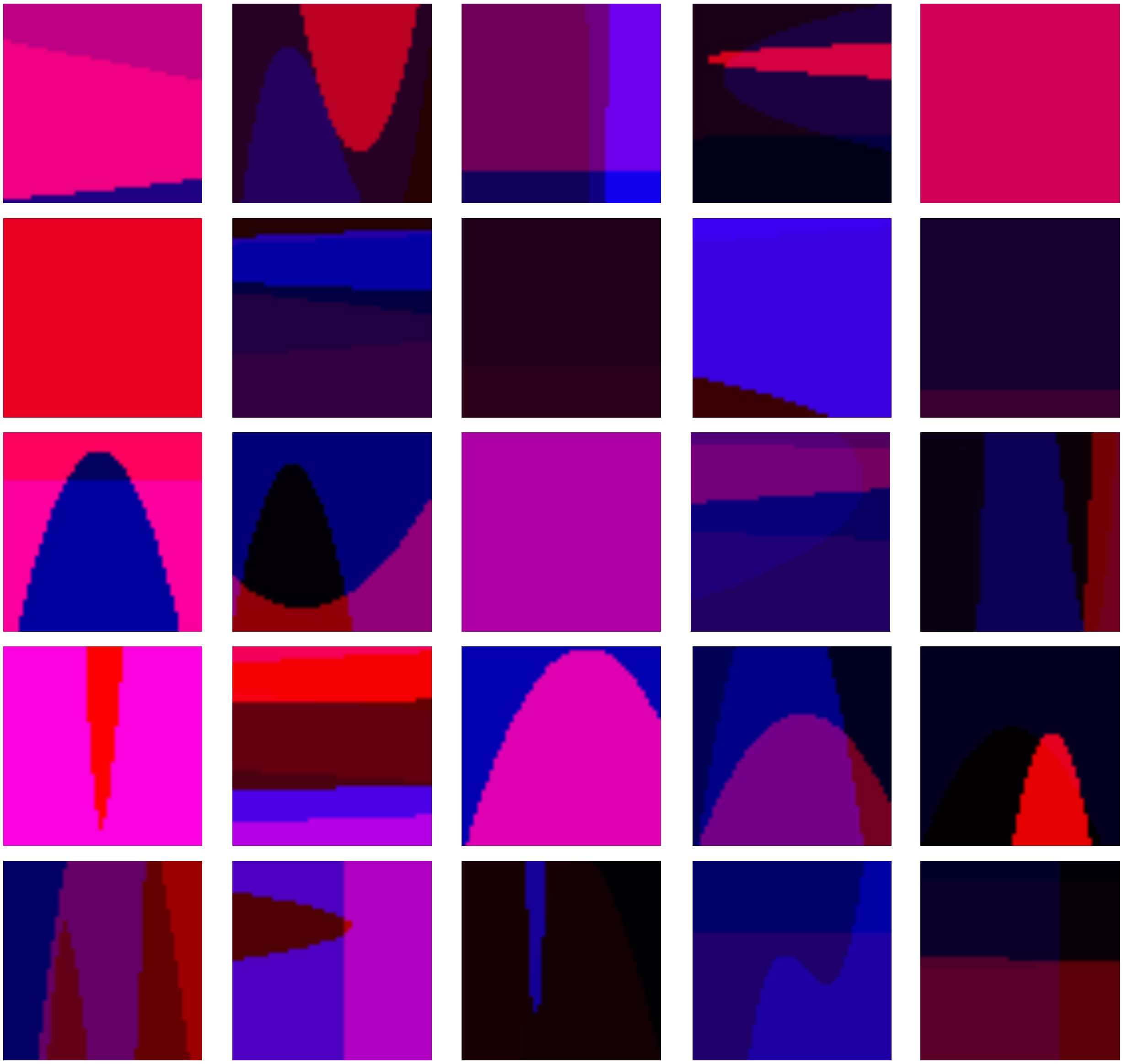

MOLECULAR ECOLOGY RESOURCES

Figure S2. Same diagram as Figure 1 from the main text, with extended caption for descriptions and output sizes of each layer. Visualized tensor sizes are proportional to the cube root of actual dimensions if $s = 5{,}000$ SNPs, $k = 10$ pairs, and $w = 10$ map width were used.

- **G1**. (s, 2) Genotypes for a pair of individuals. This branch of the network will be repeated for multiple pairs.
- **G2**. (s, 64) 1D convolution, kernel size 2, 64 filters, rectified linear unit (ReLU) activation.
- **G3**. (s/10, 64) 1D average pooling, window size 10.
- **G4**. (s/10, 108) 1D convolution, kernel size 2, 64 filters, ReLU.
- **G5**. (s/100, 108) 1D average pooling, window size 10.
- **G6**. (108s/100) Flatten.
- **G7**. (128) Dense, 128 filters, ReLU.
- **G8**. (128, k) Outputs from looping over pairs (only five pairs are shown).
- **G9**. (kw^2, 128) The outputs from k pairs are stacked together, and then duplicated for each of w^2 grid cells.
- **L1**. (kw^2, 7) Locations table for every combination of grid cell and genotype-pair (not all rows are shown).
- **L2**. (kw^2, 128) Dense, 128 filters, ReLU.
- **L3**. (kw^2, 128) Dense, 128 filters, ReLU.
- **M1**. (kw^2, 128) Element wise multiplication between layers G9 and L3, followed by ReLU.
- **M2**. (kw^2, 64) Dense, 64 filters, ReLU.
- **M3**. (w^2, 64) 1D pooling on every k rows.
- **M4**. (w^2, 64) Dense, 64 filters, ReLU.
- **M5**. (w^2, 64) Dense, 64 filters, ReLU.
- **M6**. (w^2, 2) Dense, 2 filters (linear activation).
- **M7**. (w, w, 2) Rearrange into a stack of two maps.

MOLECULAR ECOLOGY
RESOURCES

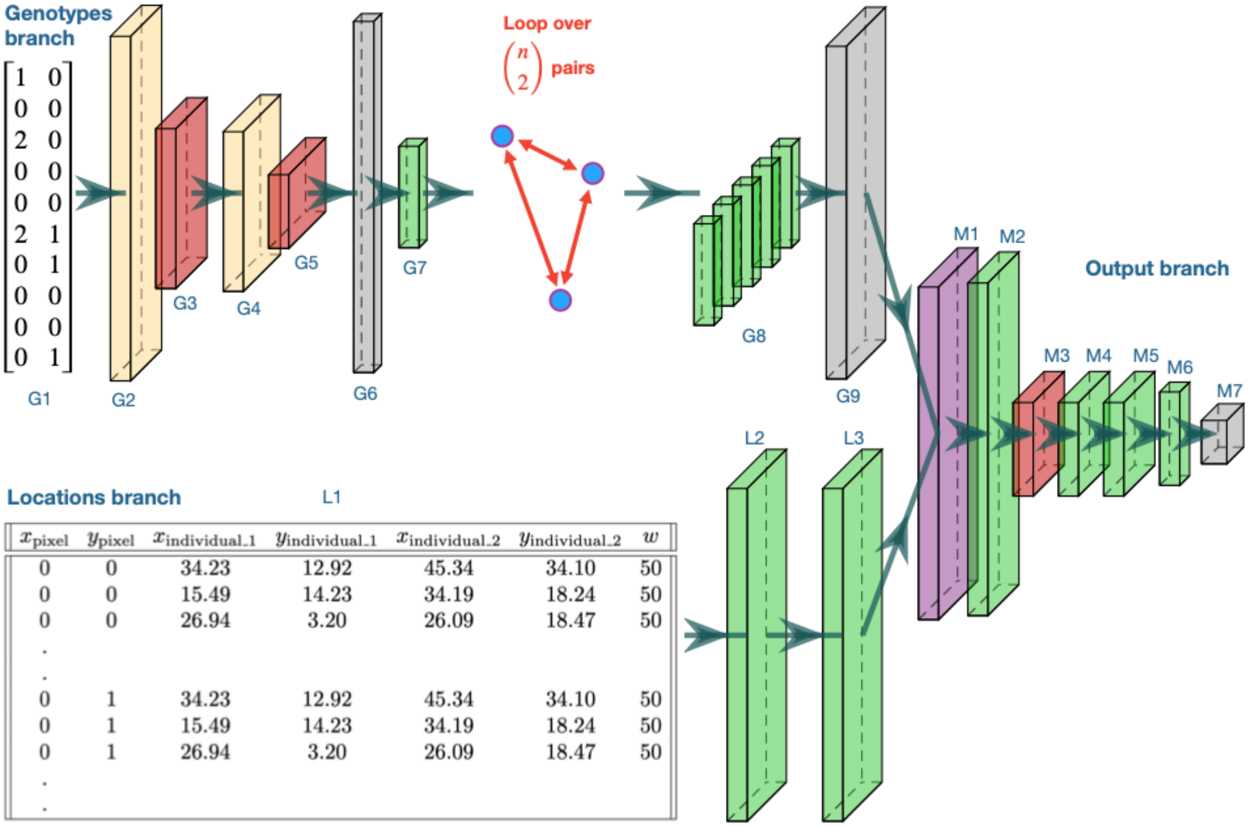

MOLECULAR ECOLOGY RESOURCES

Figure S3. The minimum dispersal rate (*σ*) supporting a stable population for different carrying capacity (*K*) values.

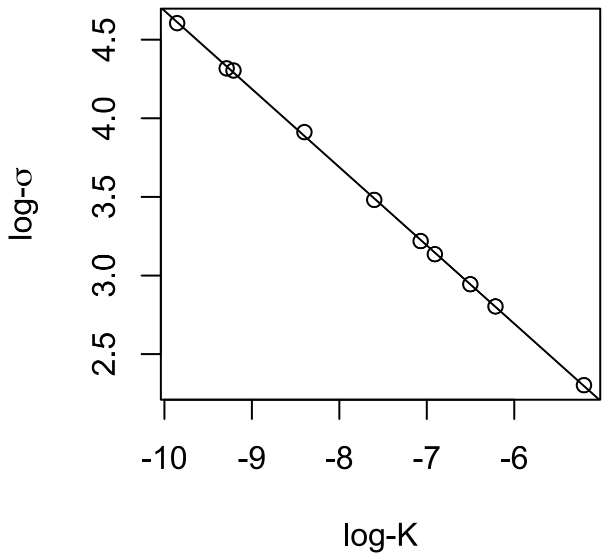

MOLECULAR ECOLOGY RESOURCES

Figure S4. PNG renderings for a random selection of training maps for the North American grey wolf analysis. The blue channel conveys dispersal rate and the red channel conveys carrying capacity.

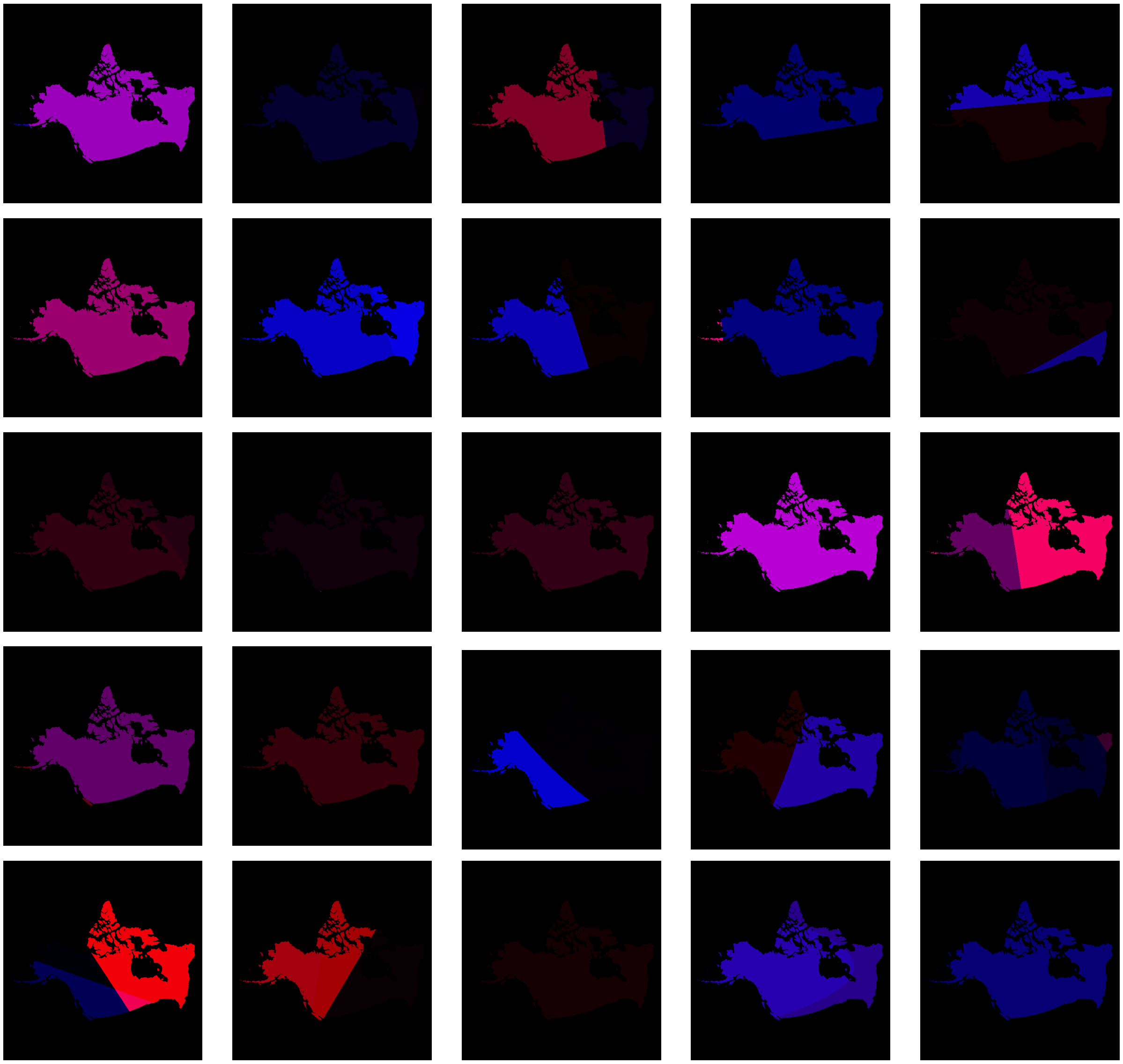

MOLECULAR ECOLOGY RESOURCES

Figures S5-S9. Predicted maps for a randomly selected, simulated test dataset. The leftmost column shows the ground truth maps for dispersal (top row) and density (bottom row). Columns 2-4 show estimated maps using three different methods: mapNN, FEEMS, and MAPS (respectively).

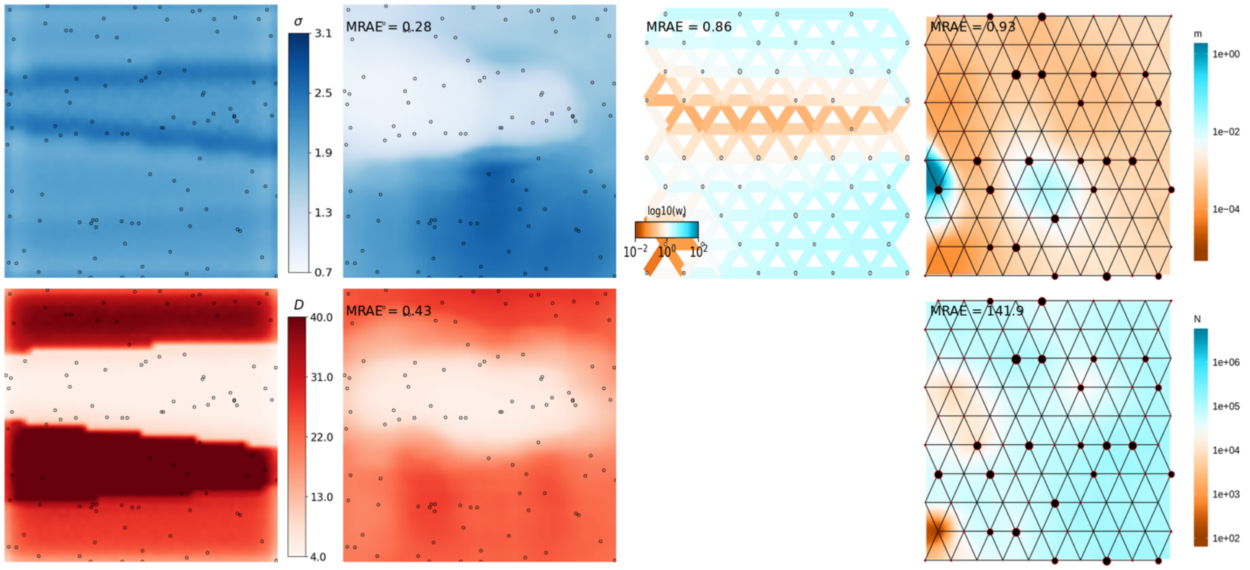

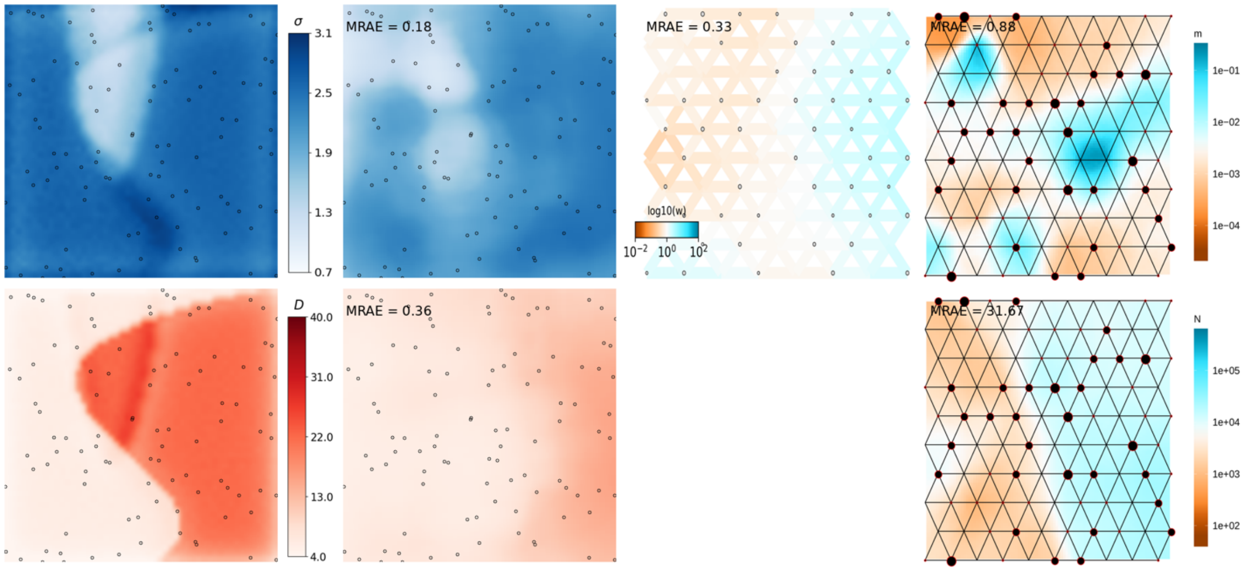

MOLECULAR ECOLOGY
RESOURCES

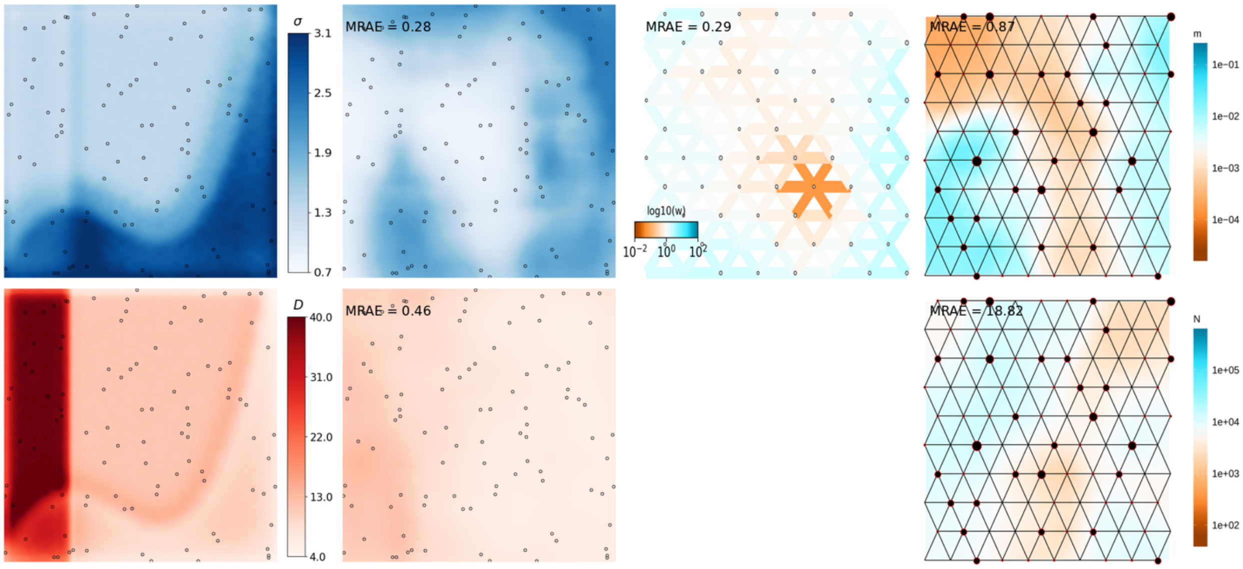

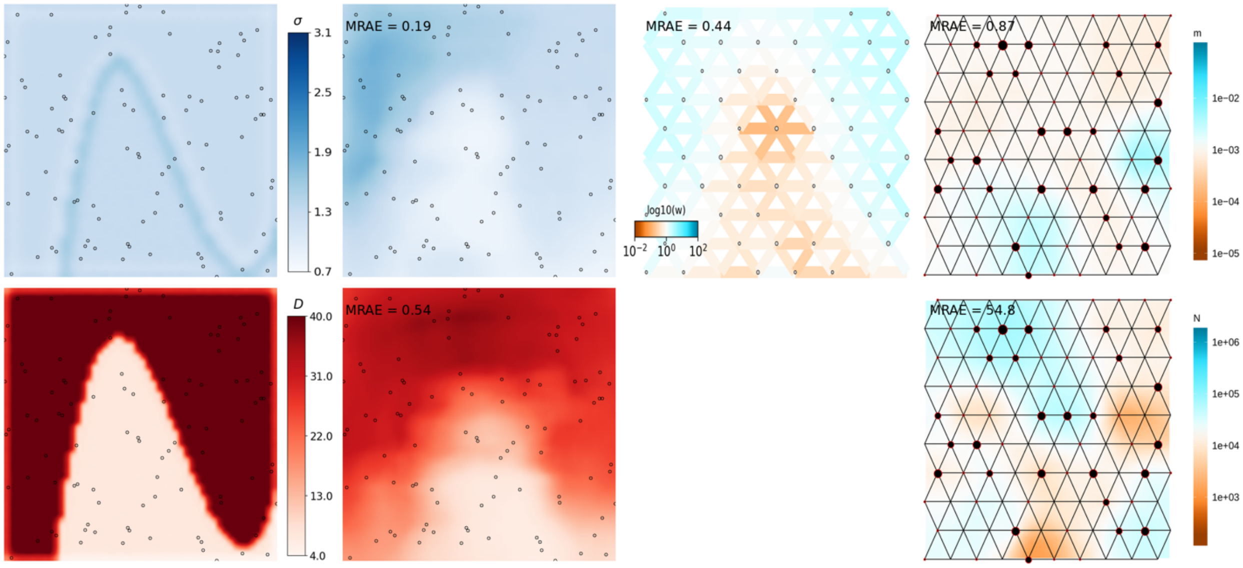

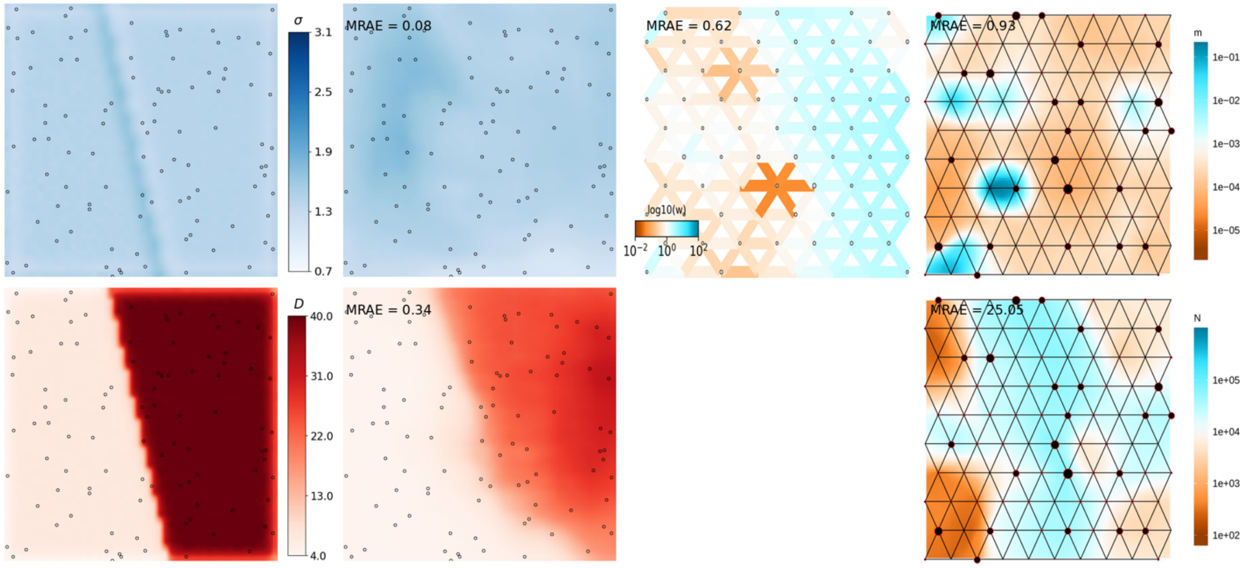
σ
3.1
2.5
1.9
1.3
0.7
MRAE = 0.08
MRAE = 0.62
log10(w)
10^{-2} 10^{0} 10^{2}
MRAE = 0.93
m
1e−01
1e−02
1e−03
1e−04
1e−05
D
40.0
31.0
22.0
13.0
4.0
MRAE = 0.34
MRAE = 25.05
N
1e+05
1e+04
1e+03
1e+02

MOLECULAR ECOLOGY RESOURCES

Figure S10-S14. Predicted maps for a randomly selected, simulated test dataset for the North American grey wolf analysis. The left column shows the ground truth maps for dispersal (top row) and density (bottom row). The right-hand column shows estimated maps from mapNN.

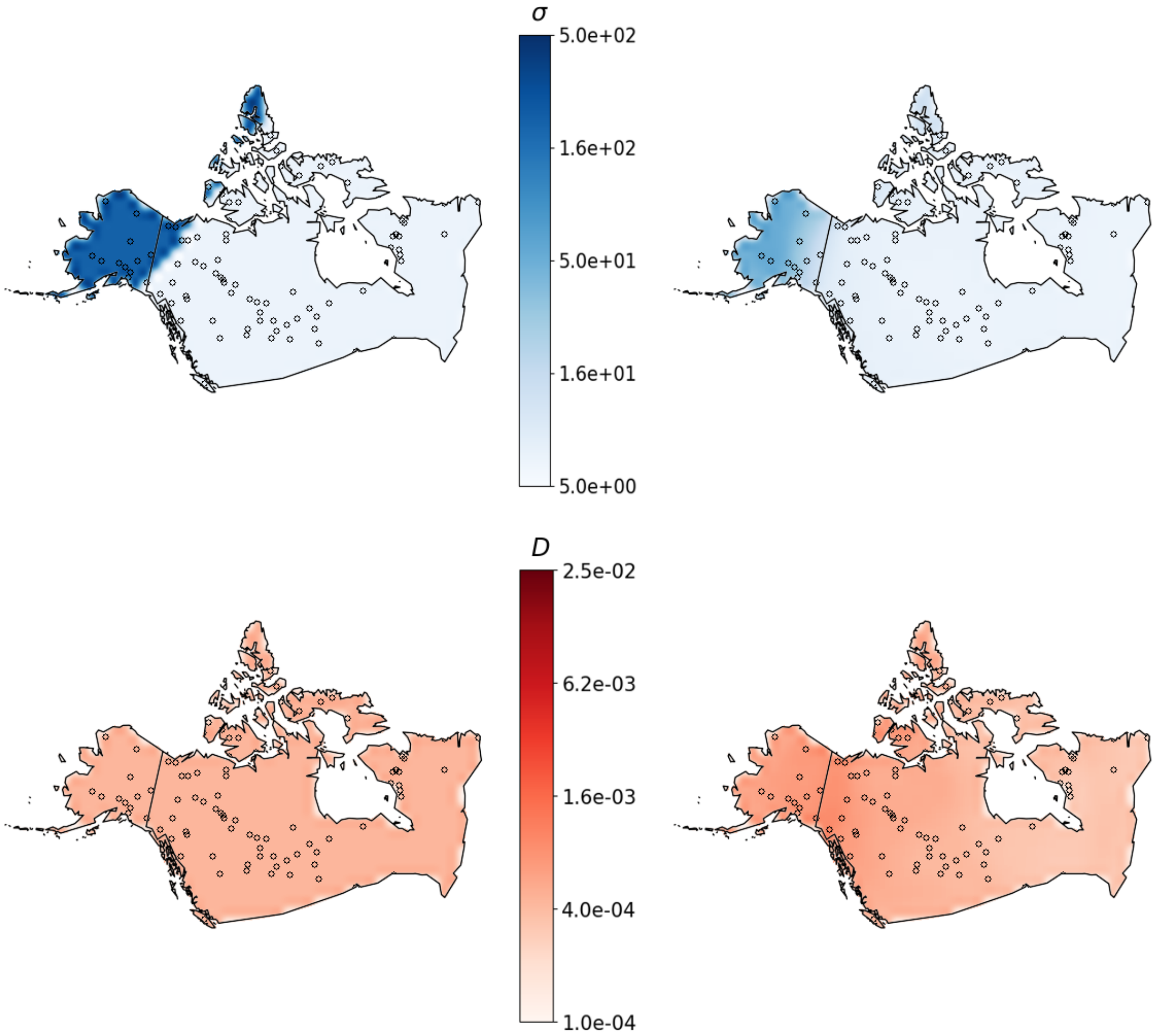

MOLECULAR ECOLOGY
RESOURCES

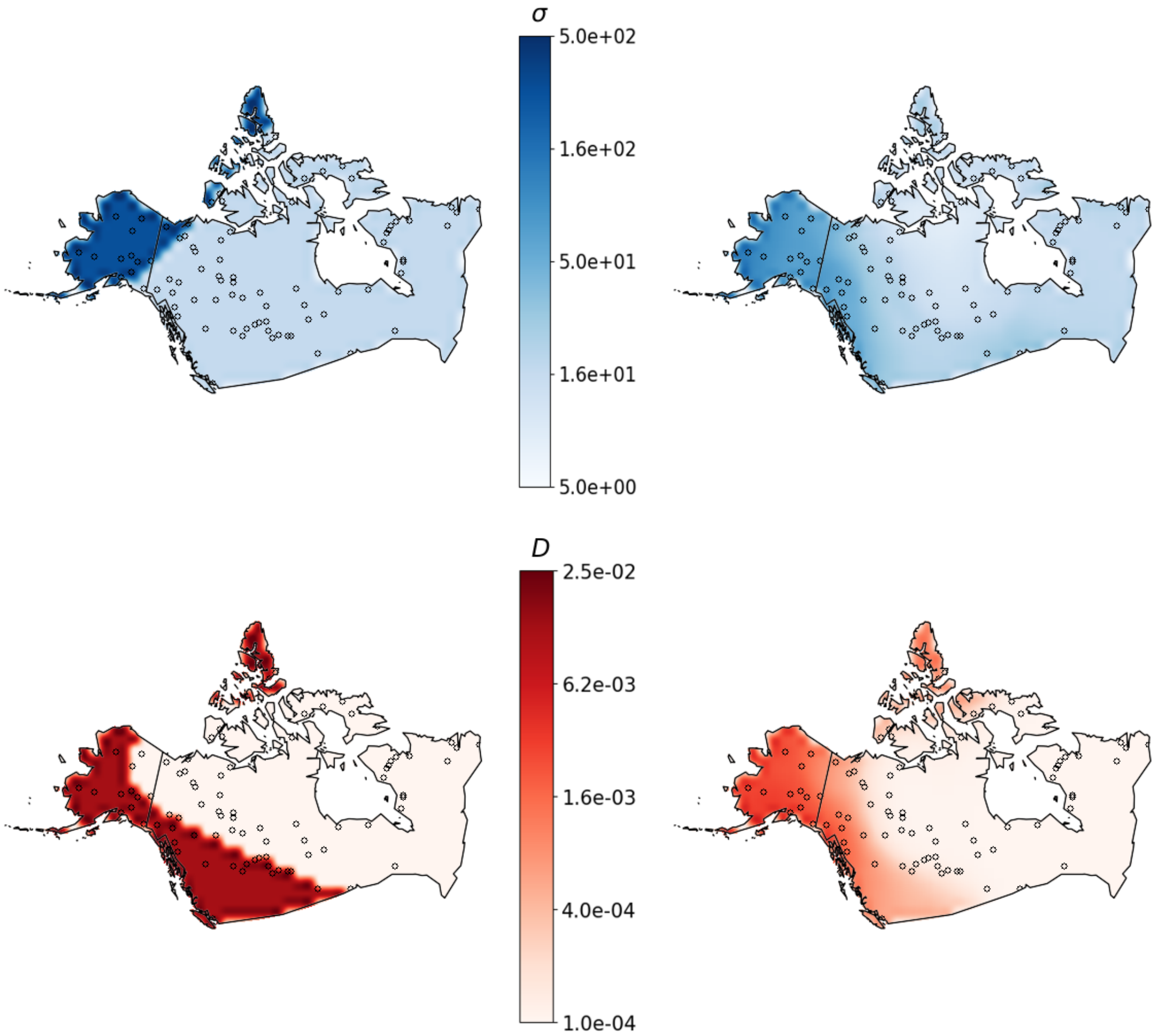

MOLECULAR ECOLOGY
RESOURCES

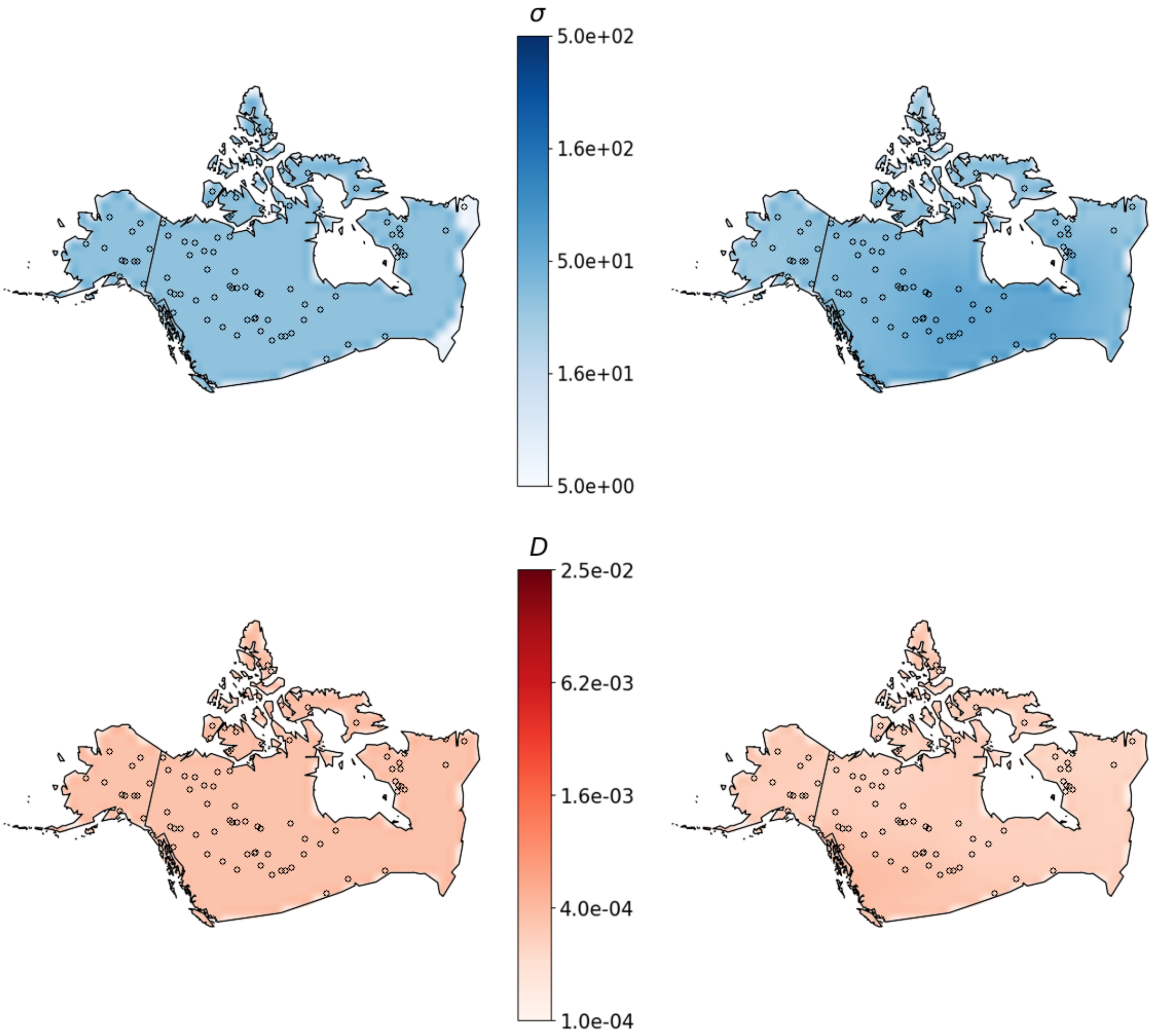

MOLECULAR ECOLOGY RESOURCES

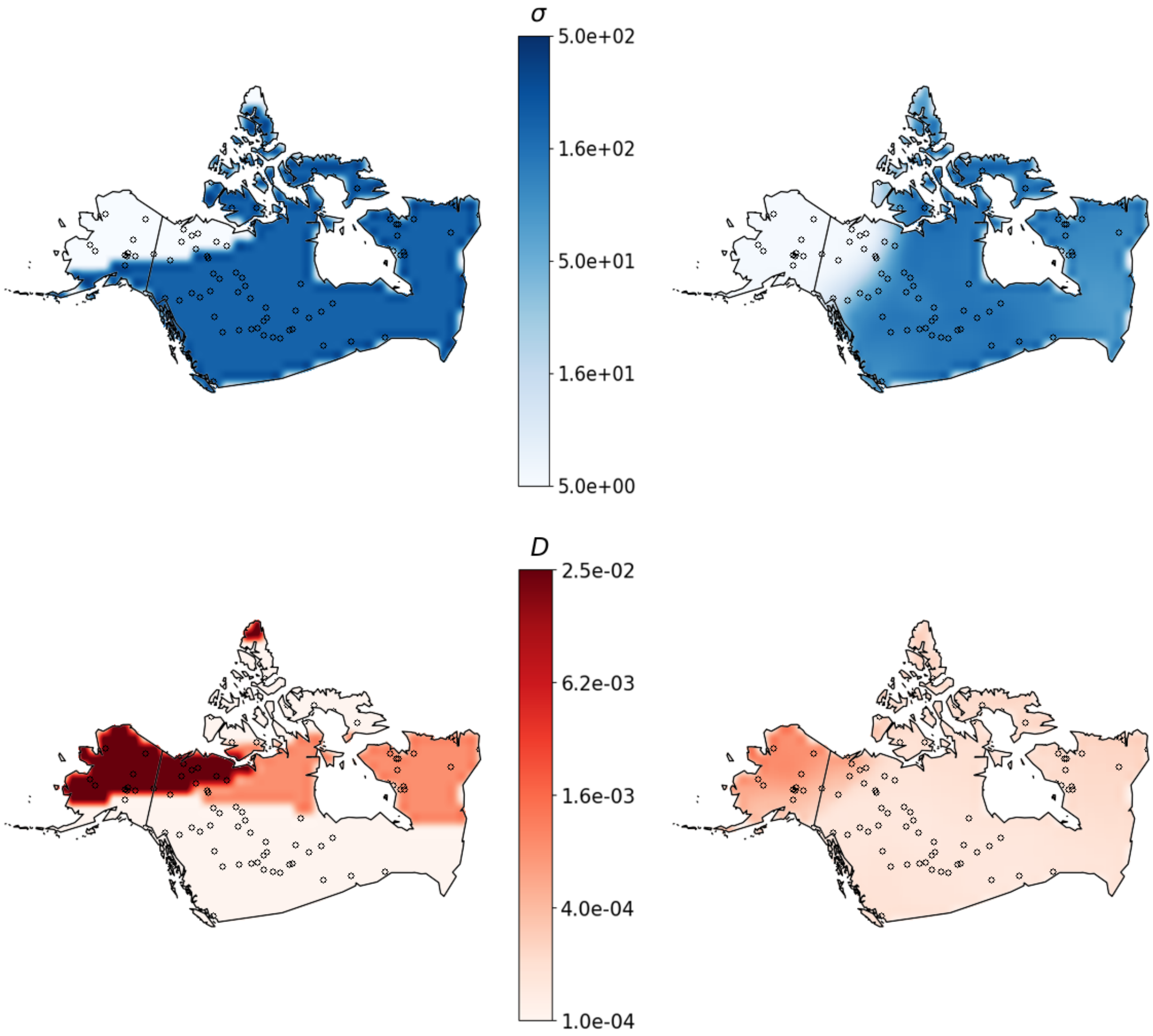

MOLECULAR ECOLOGY
RESOURCES

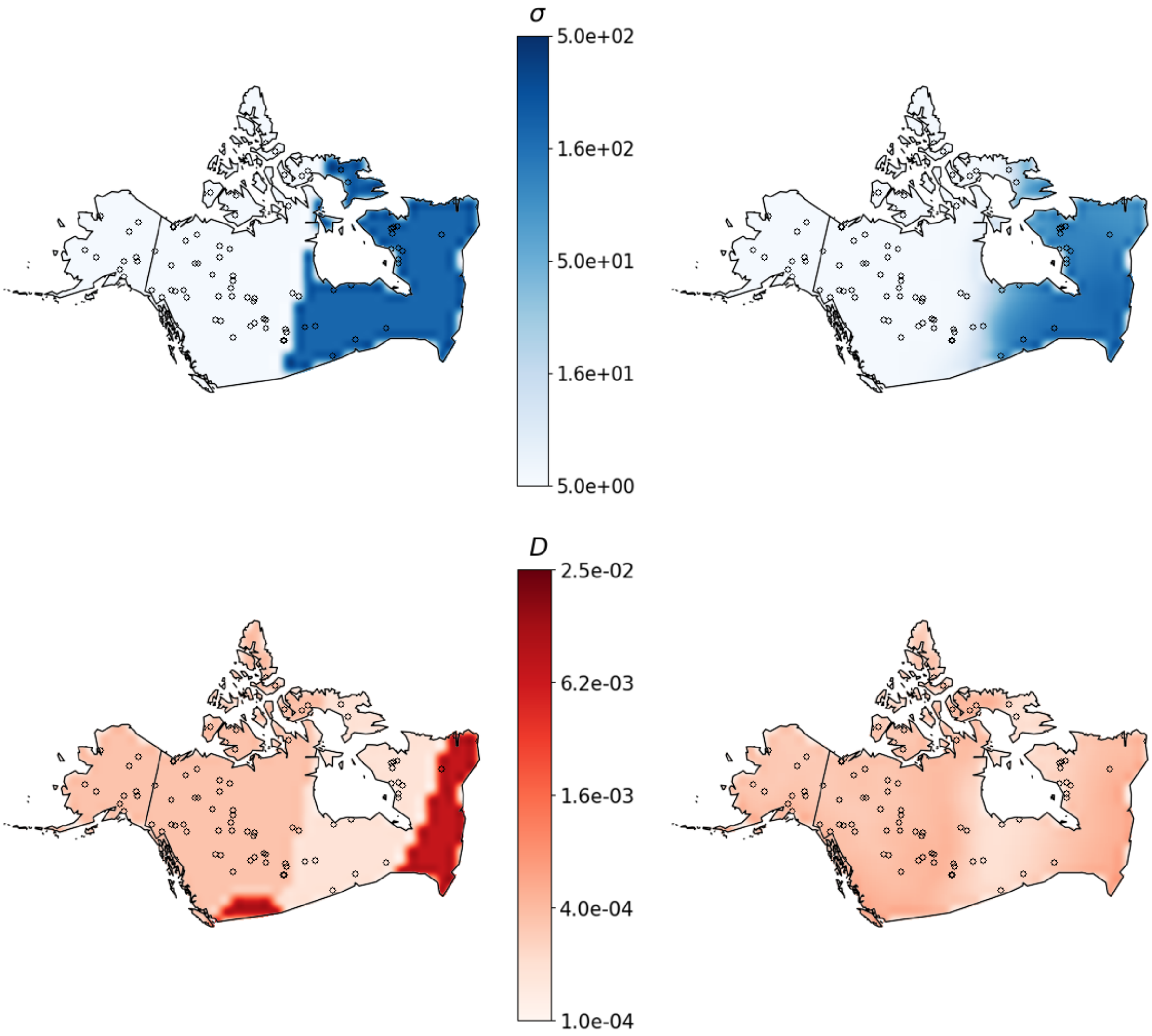

MOLECULAR ECOLOGY
RESOURCES

Figure S15. FEEMS run on the same n = 94 individuals analyzed in the current study. Analysis by Vivaswat Shastry.

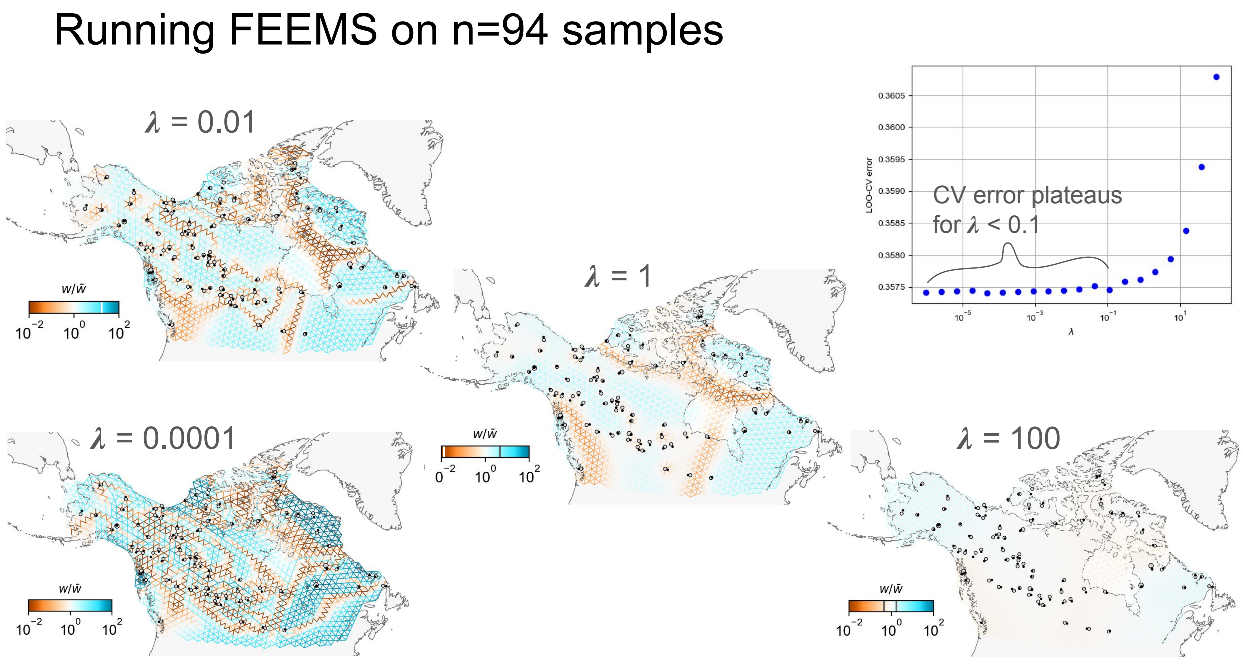

Supplement: supplement [file NIHMS2092588-supplement-supplement.zip › Supplemental_Information.pdf]
