# Supplementary figures and images for: Estimation of spatial demographic maps from polymorphism data using a neural network
Source: Mol Ecol Resour. Author manuscript; Available in PMC 2025 Jul 20. (PMC12276964; doi:10.1111/1755-0998.14005)

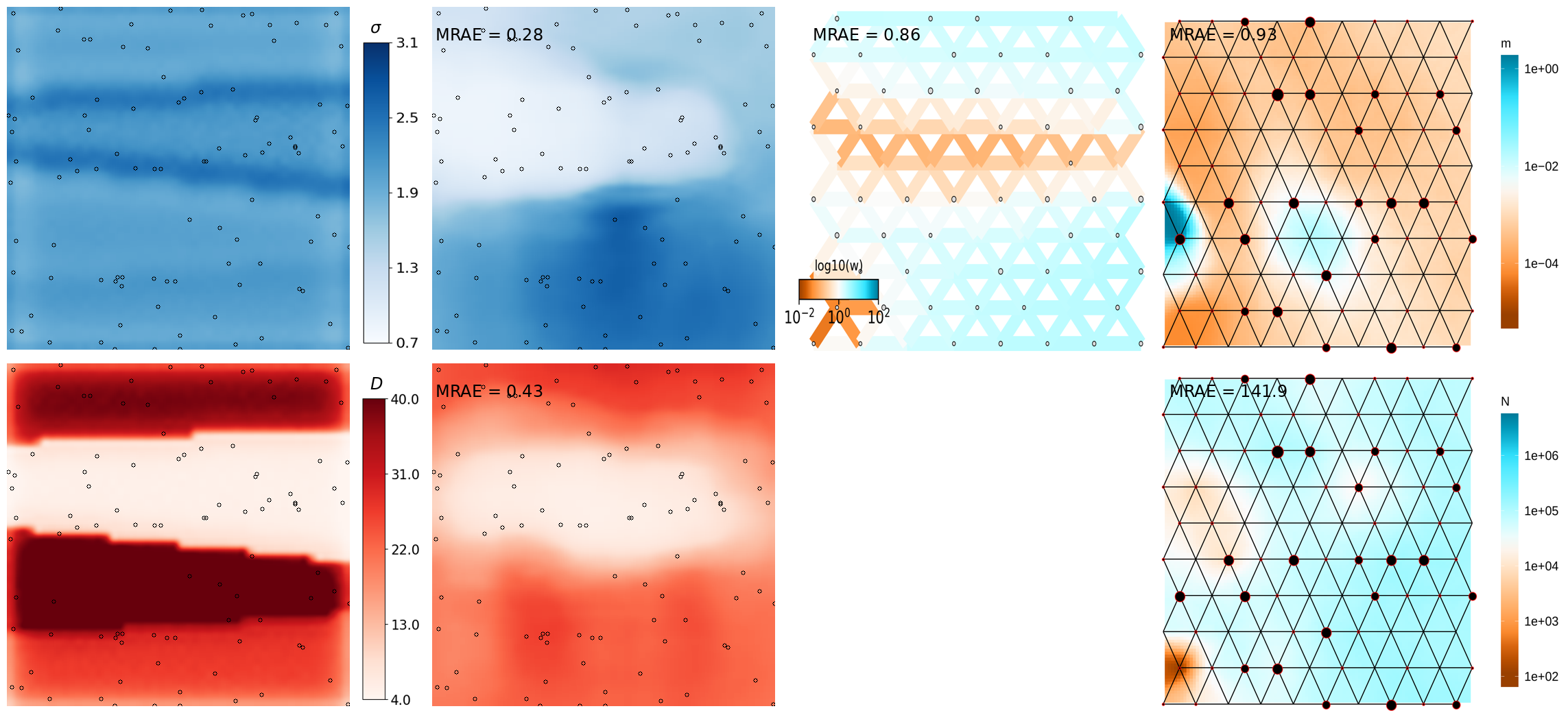

Supplement: supplement [file NIHMS2092588-supplement-supplement.zip › output_1.png]

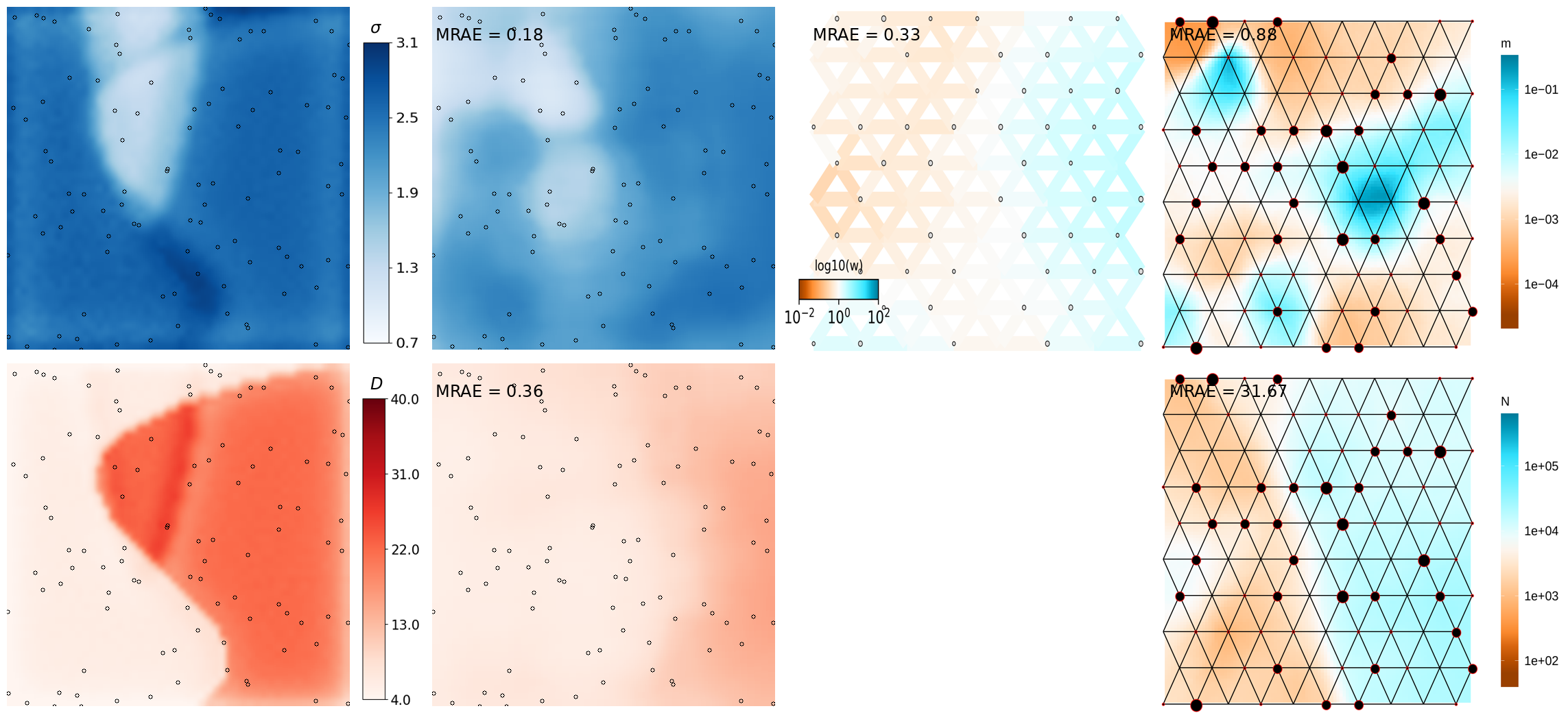

Supplement: supplement [file NIHMS2092588-supplement-supplement.zip › output_2.png]

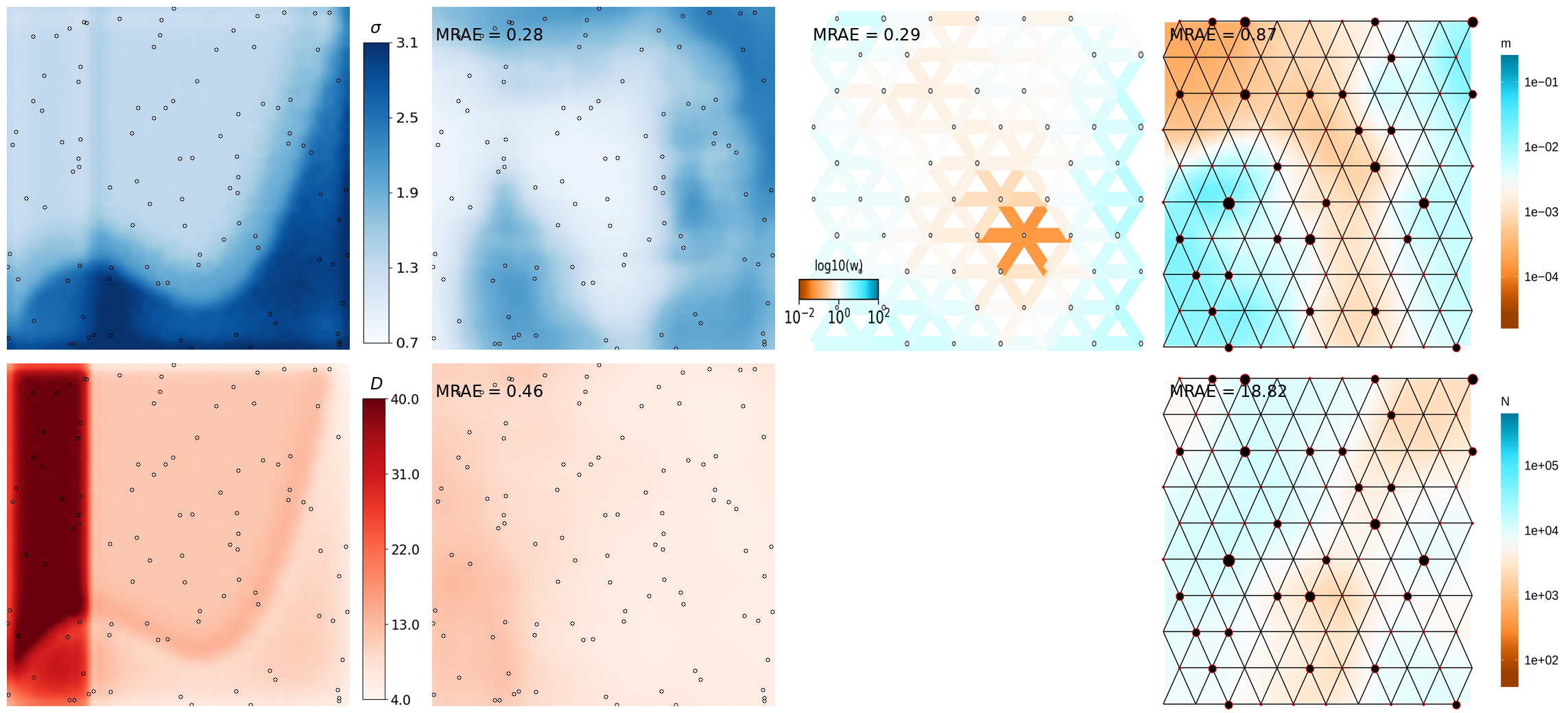

Supplement: supplement [file NIHMS2092588-supplement-supplement.zip › output_3.png]

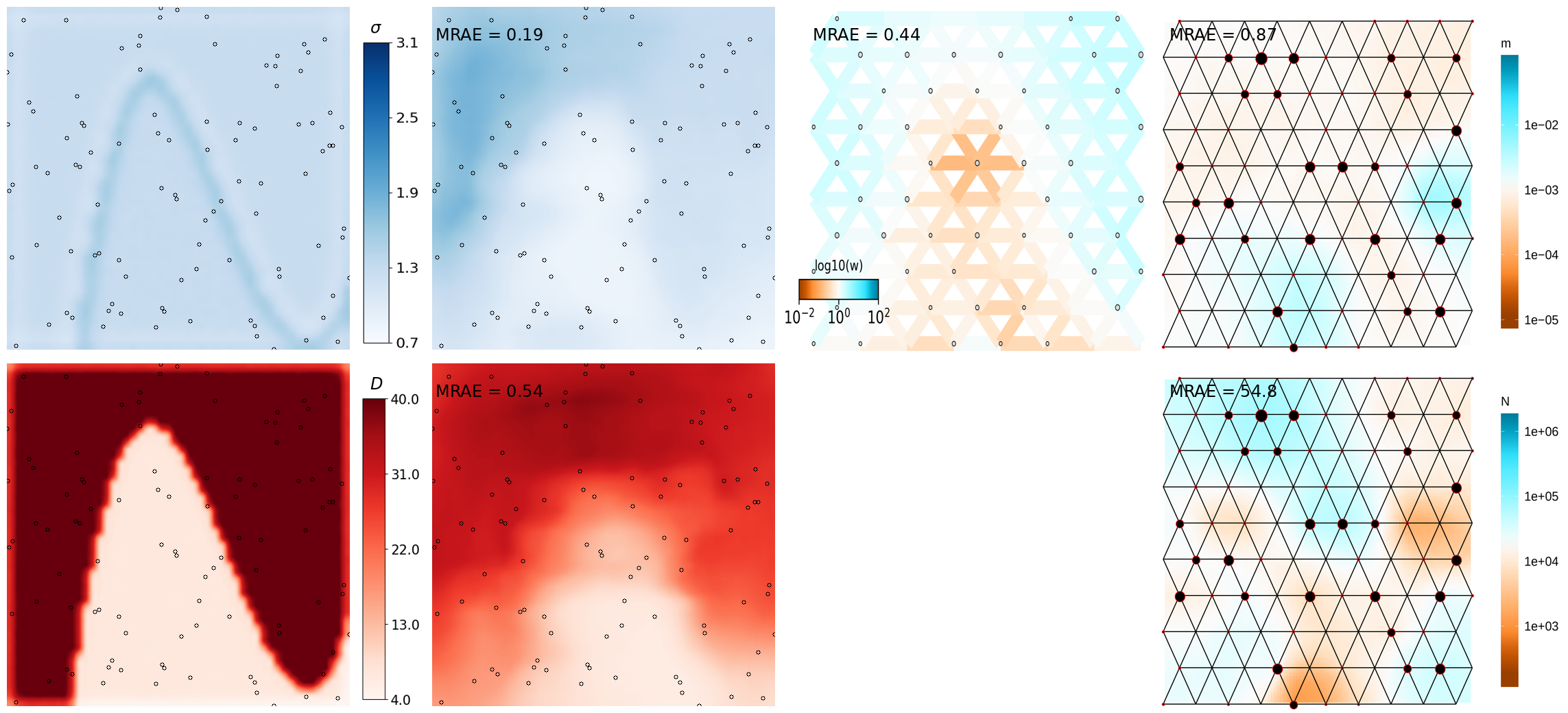

Supplement: supplement [file NIHMS2092588-supplement-supplement.zip › output_4.png]

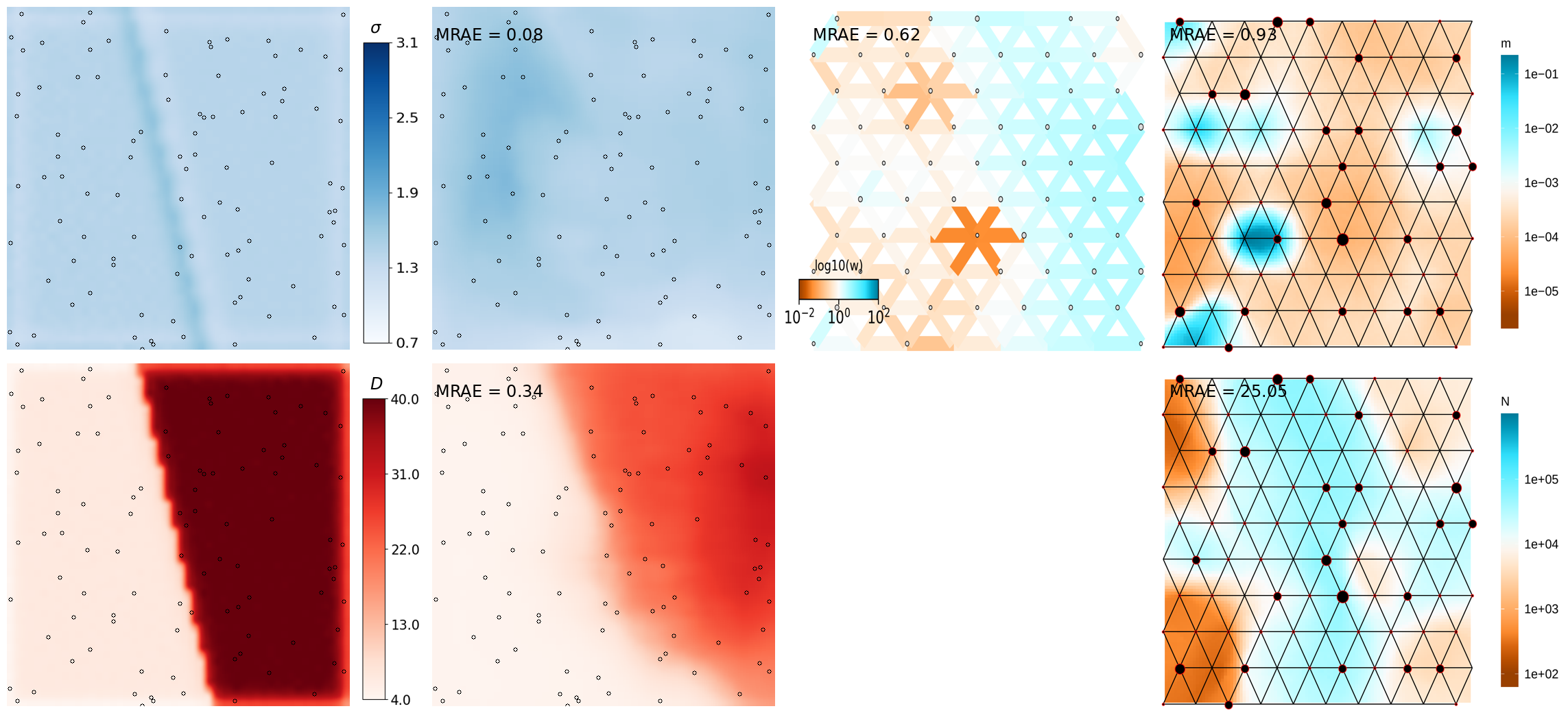

Supplement: supplement [file NIHMS2092588-supplement-supplement.zip › output_5.png]

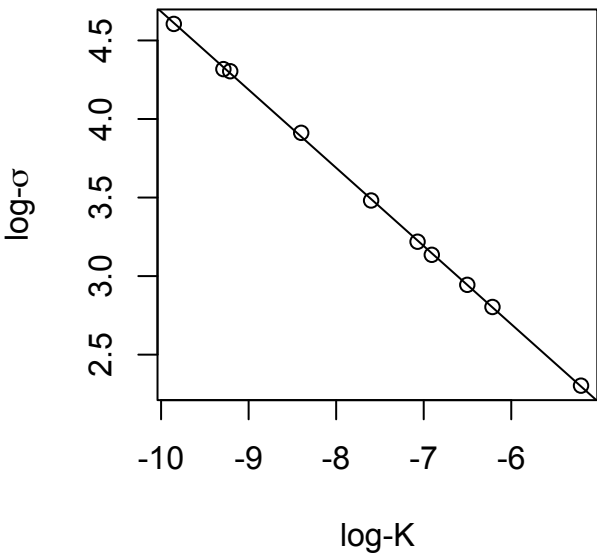

Supplement: supplement [file NIHMS2092588-supplement-supplement.zip › prior_correction.pdf]

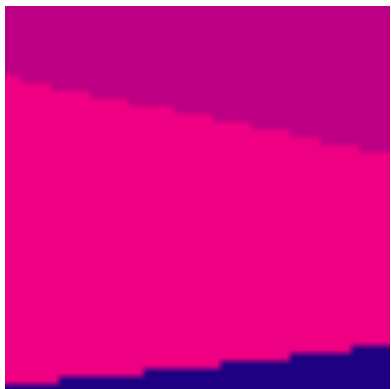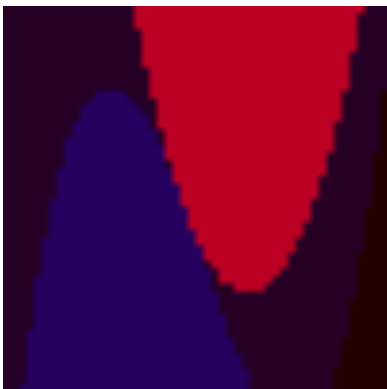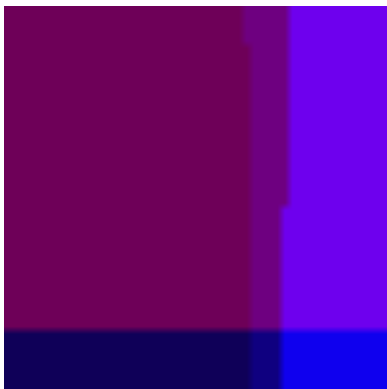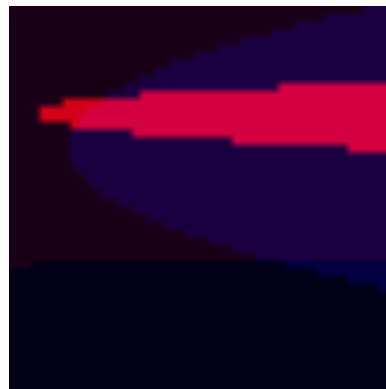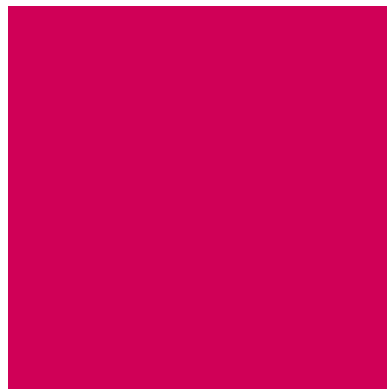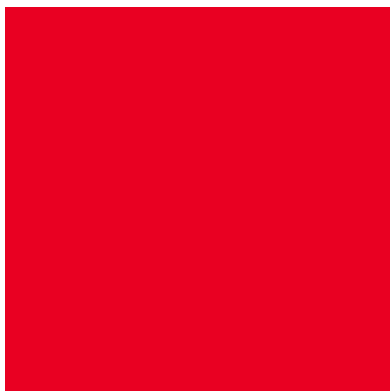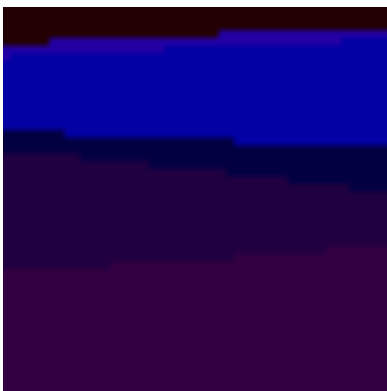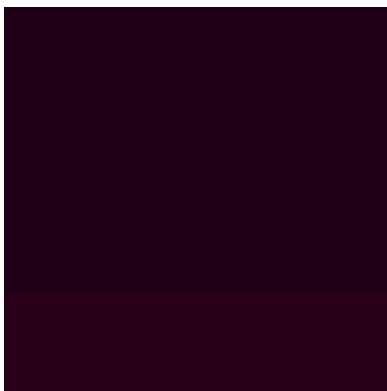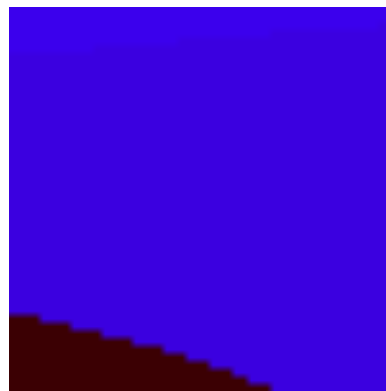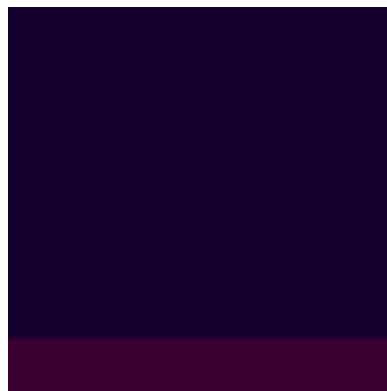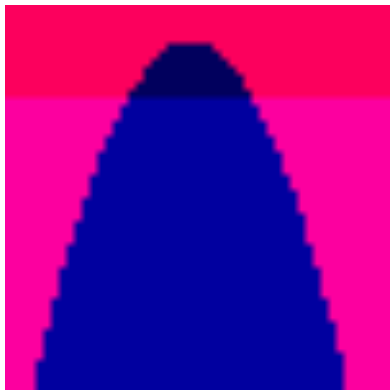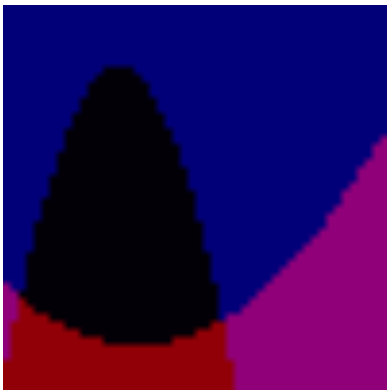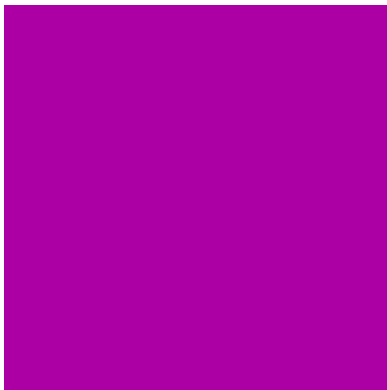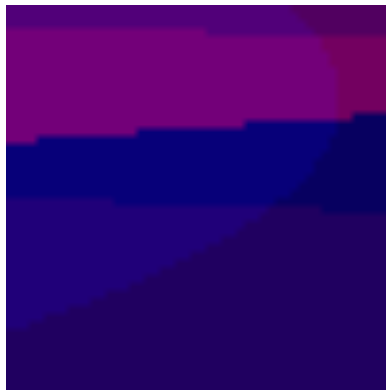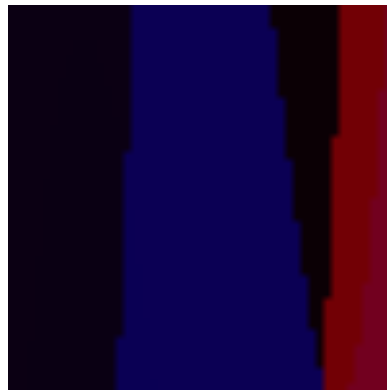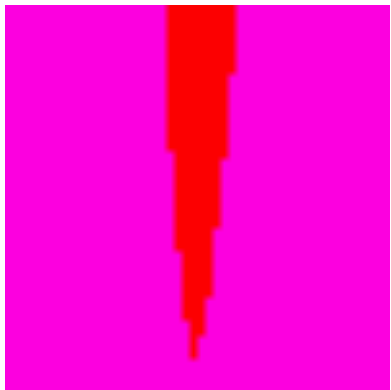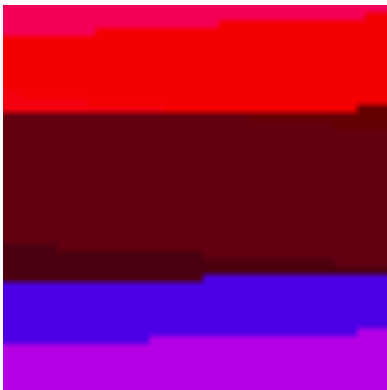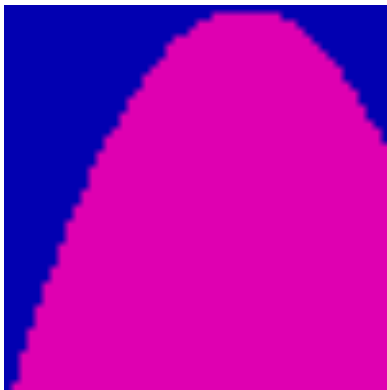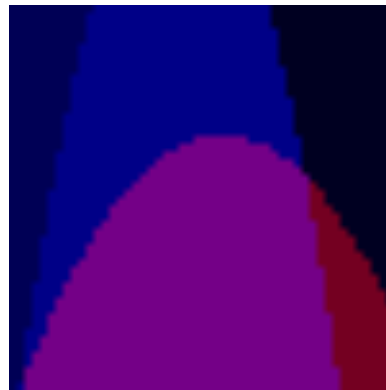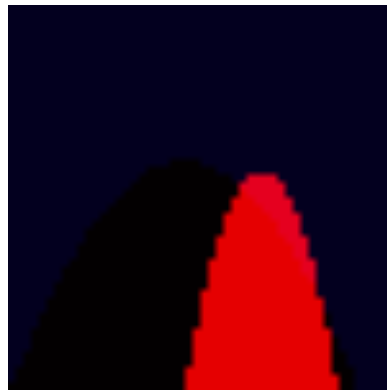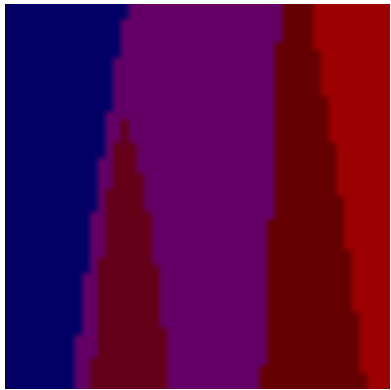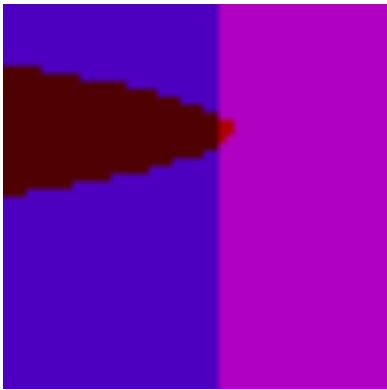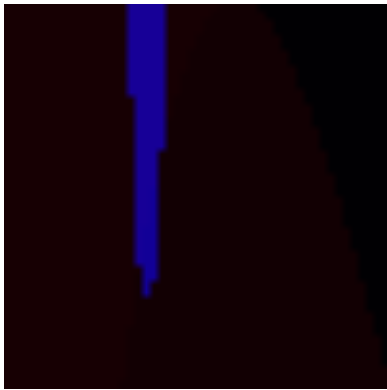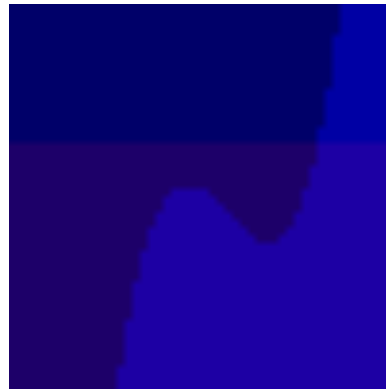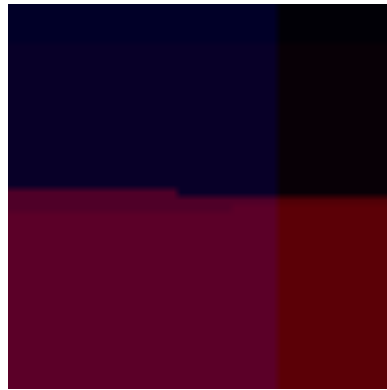

Supplement: supplement [file NIHMS2092588-supplement-supplement.zip › pump_up_the_jams.pdf]

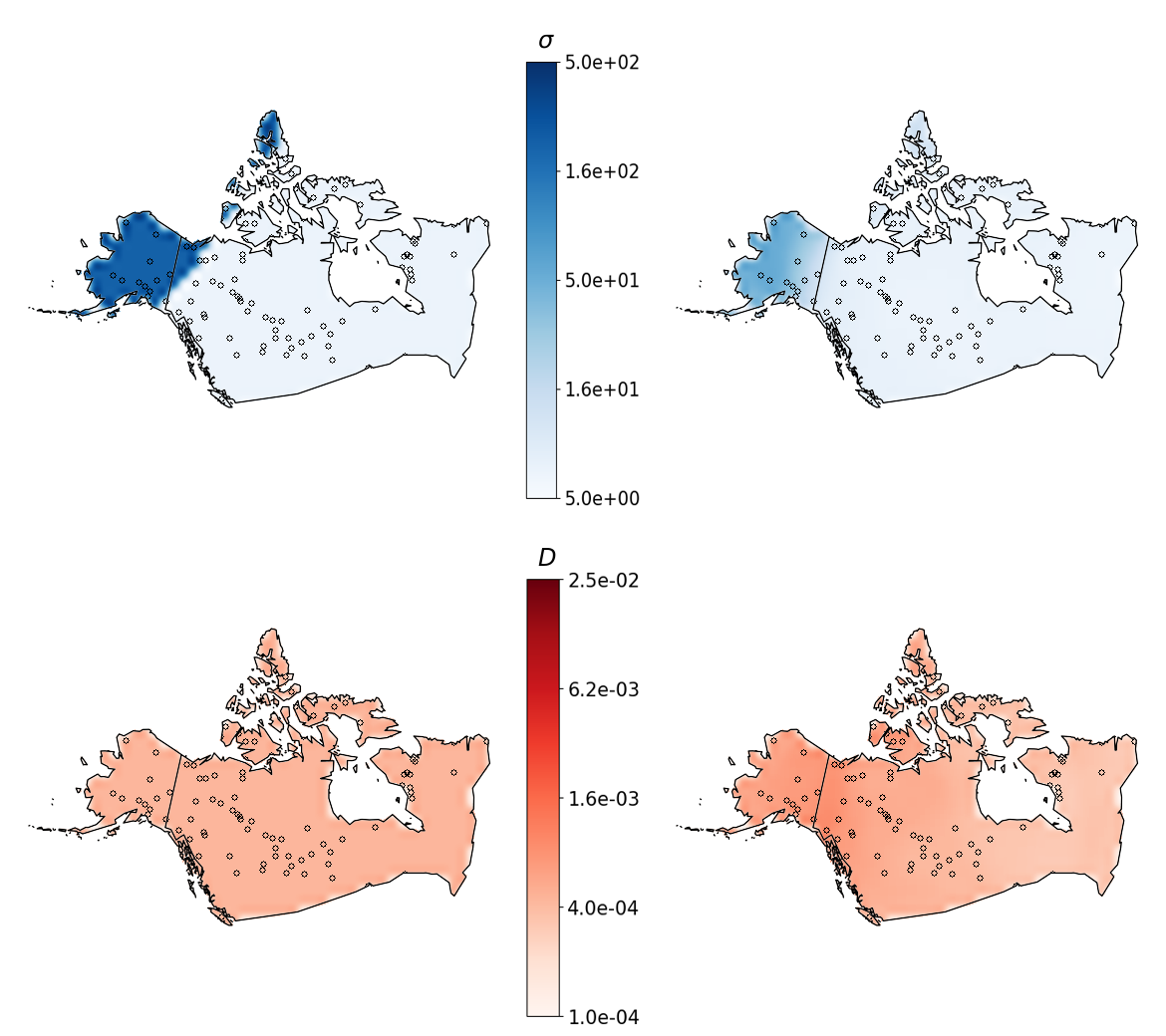

Supplement: supplement [file NIHMS2092588-supplement-supplement.zip › wolf_1.png]

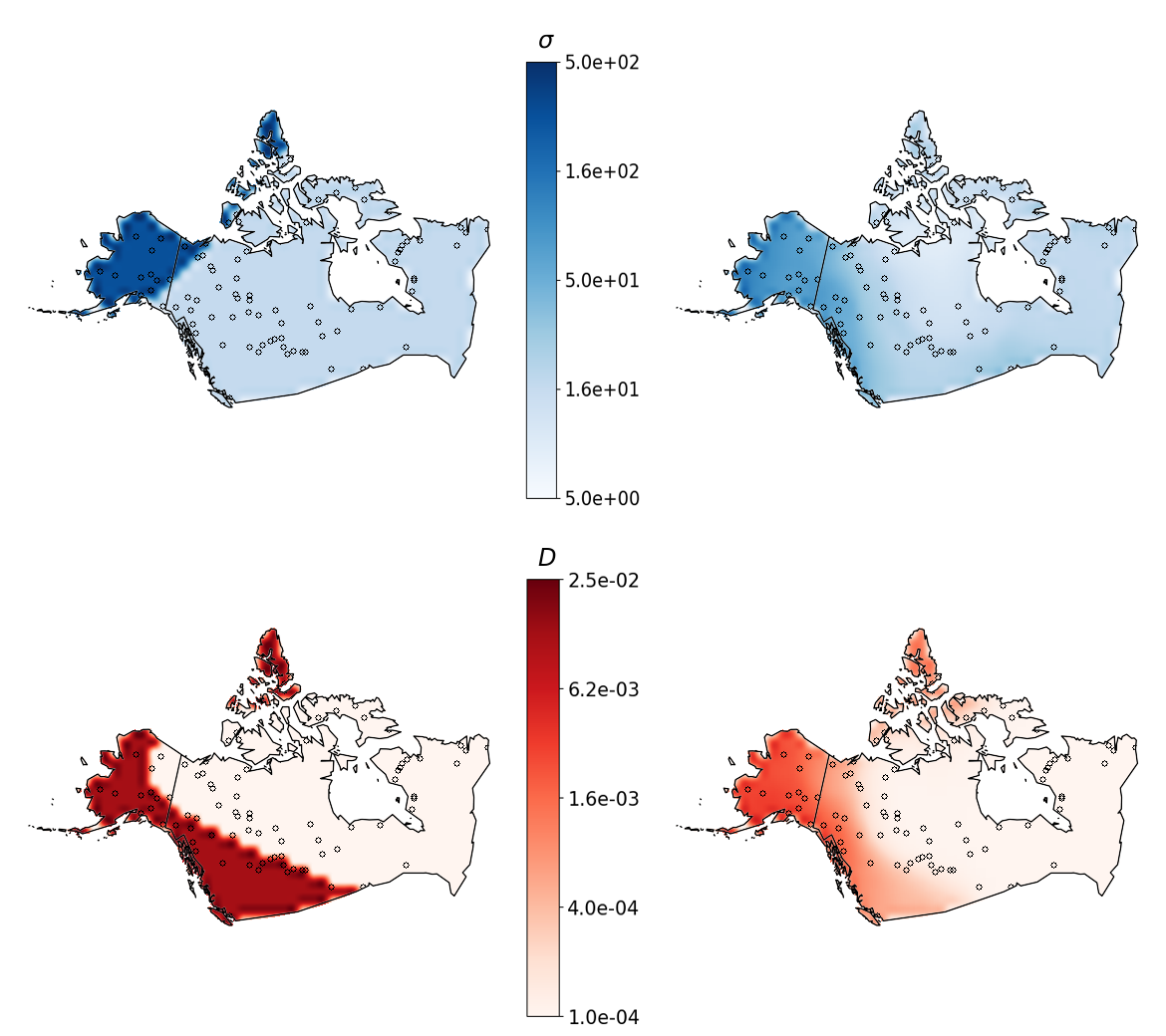

Supplement: supplement [file NIHMS2092588-supplement-supplement.zip › wolf_2.png]

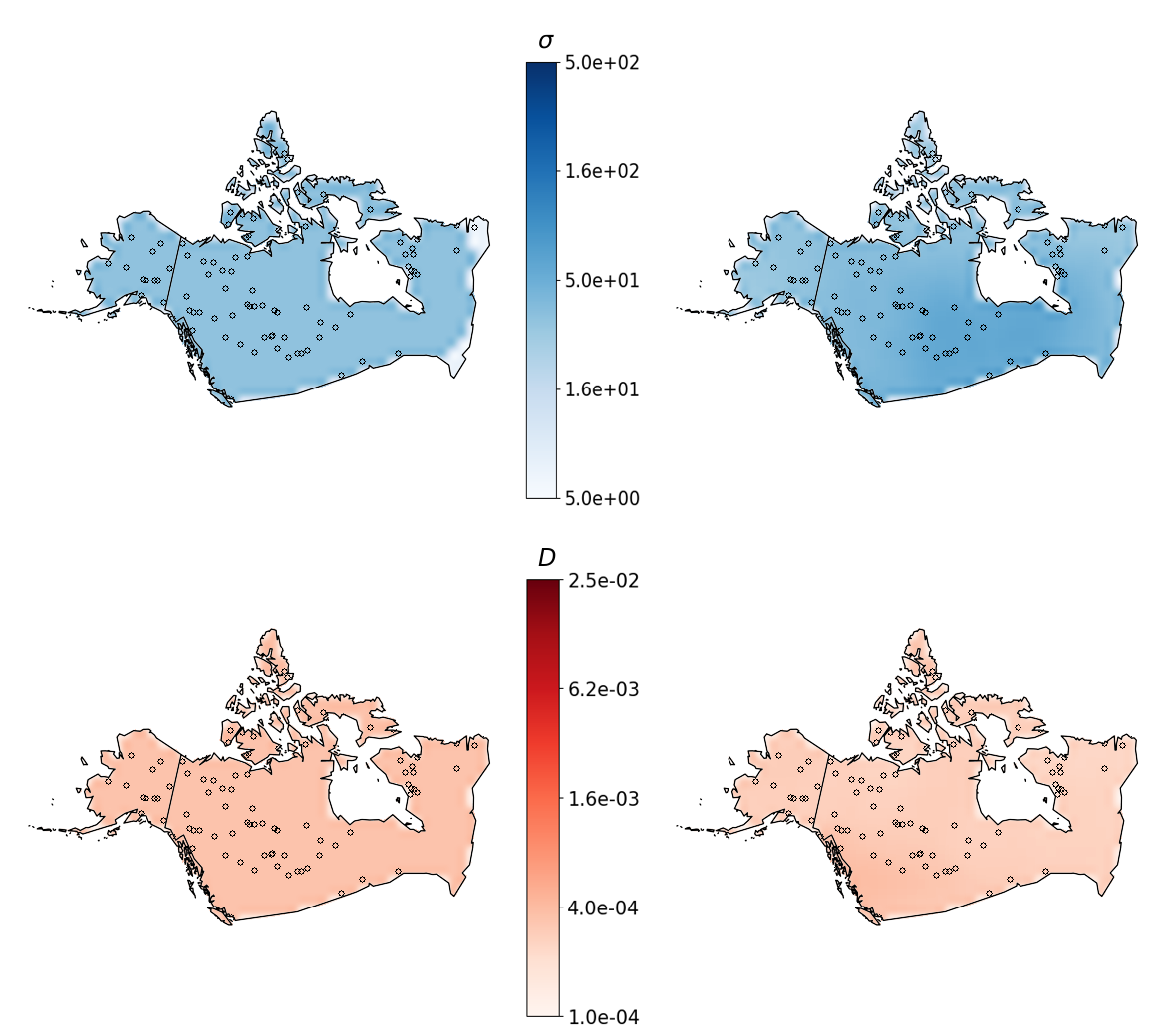

Supplement: supplement [file NIHMS2092588-supplement-supplement.zip › wolf_3.png]

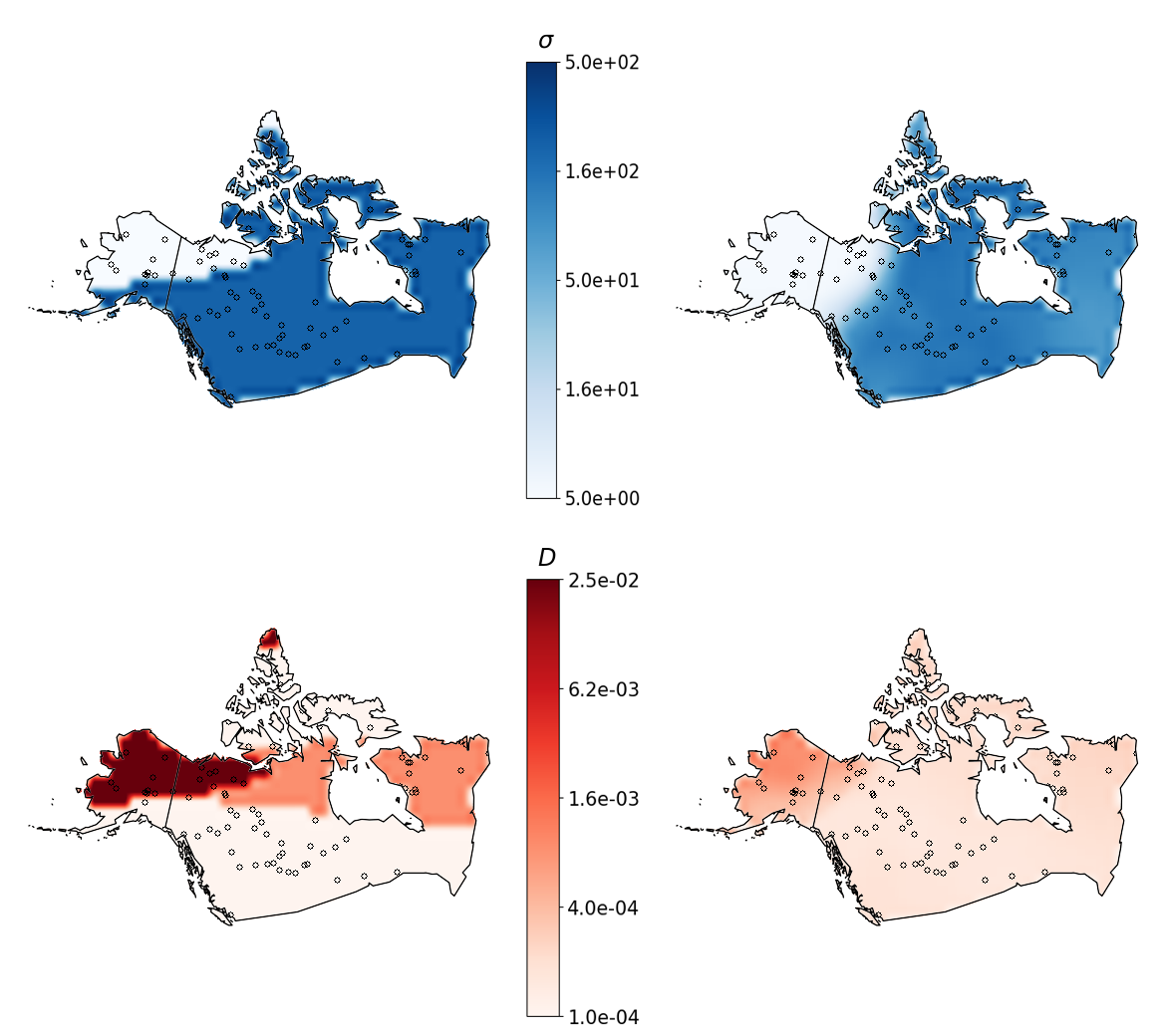

Supplement: supplement [file NIHMS2092588-supplement-supplement.zip › wolf_4.png]

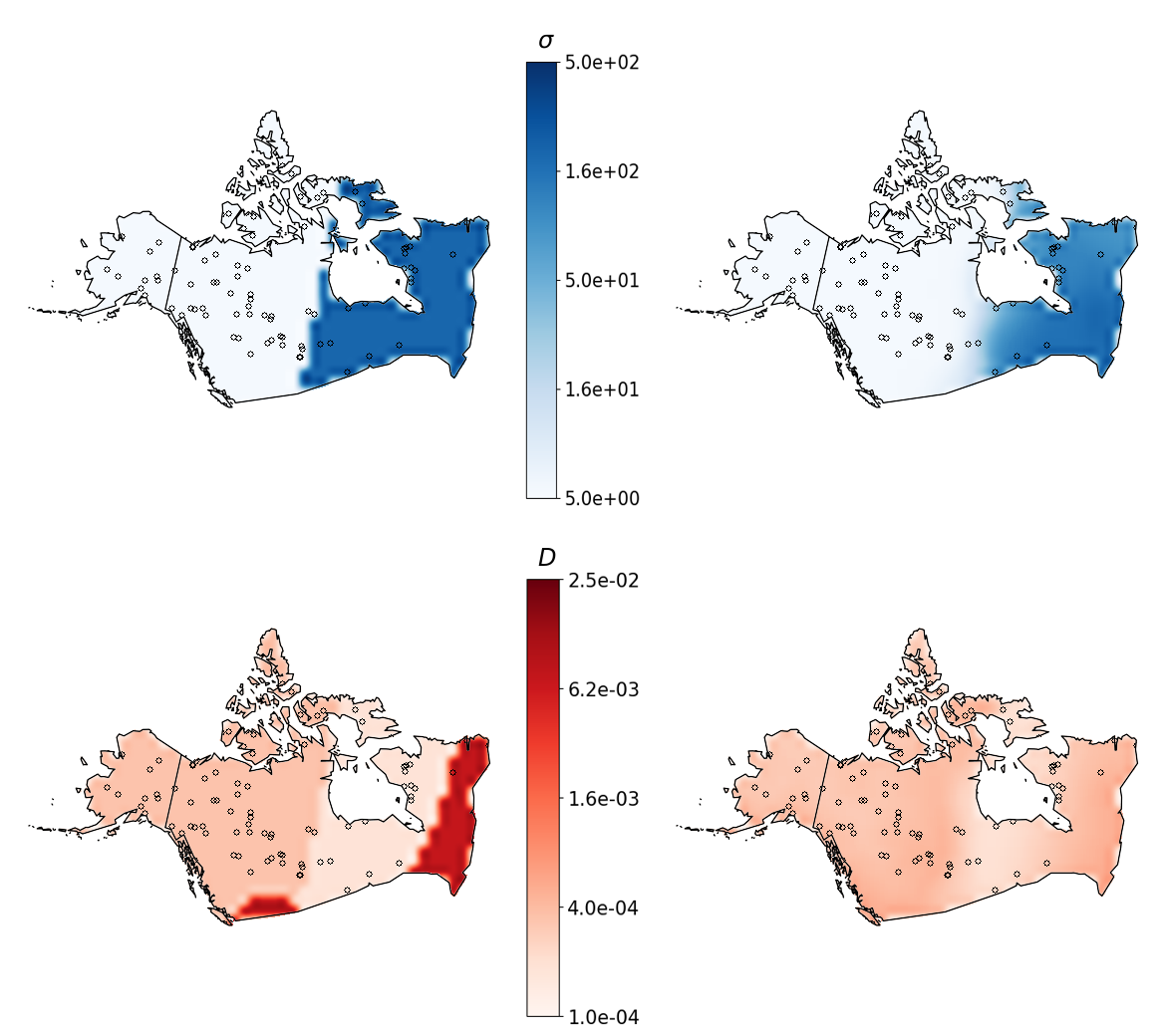

Supplement: supplement [file NIHMS2092588-supplement-supplement.zip › wolf_5.png]

# Running FEEMS on n=94 samples

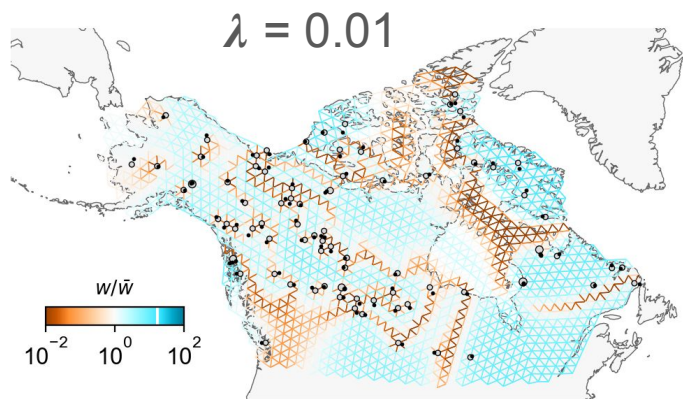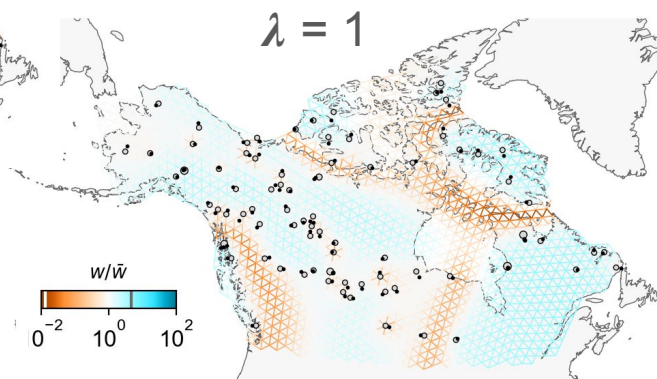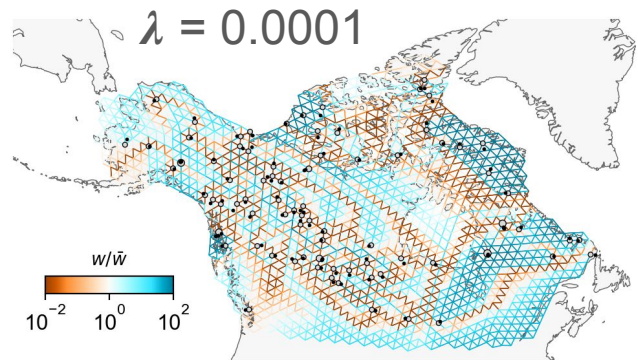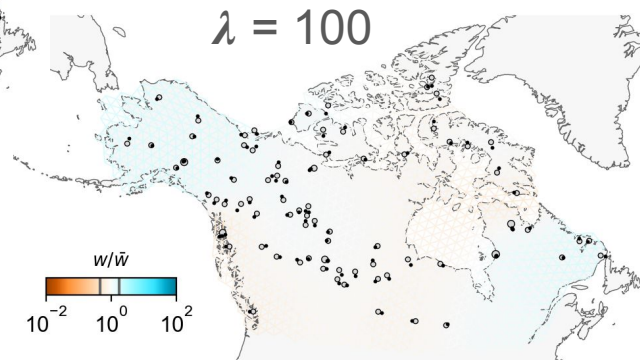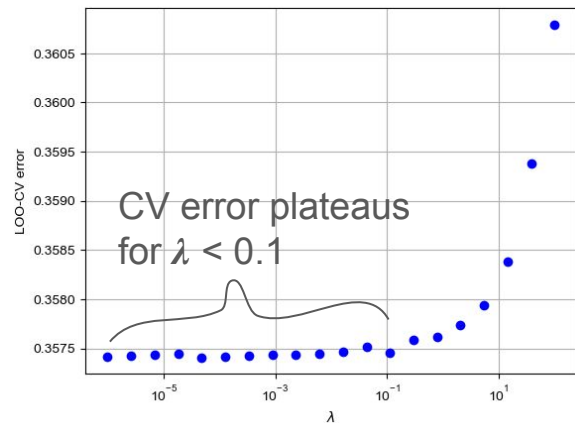

Supplement: supplement [file NIHMS2092588-supplement-supplement.zip › wolf_feems.pdf]

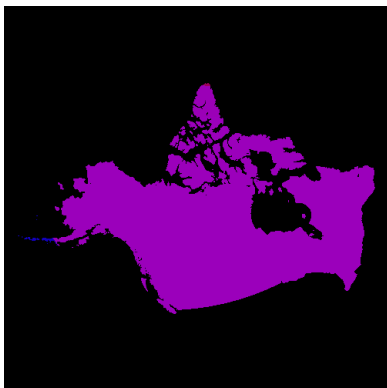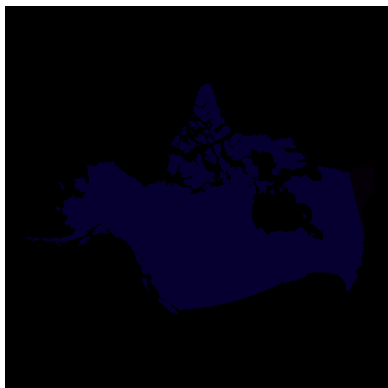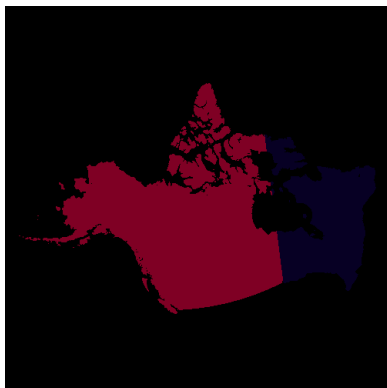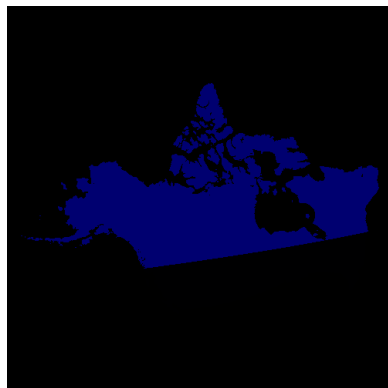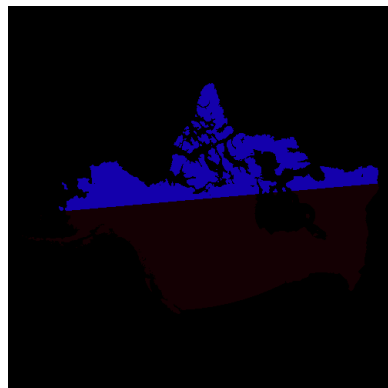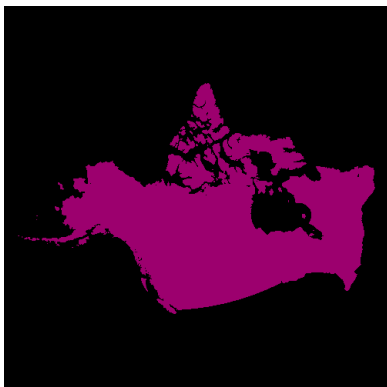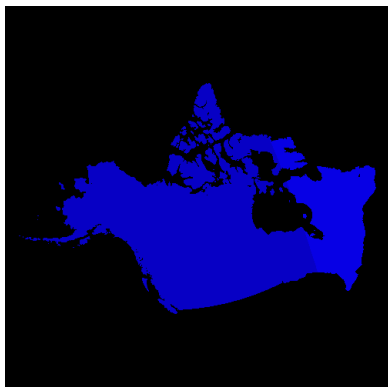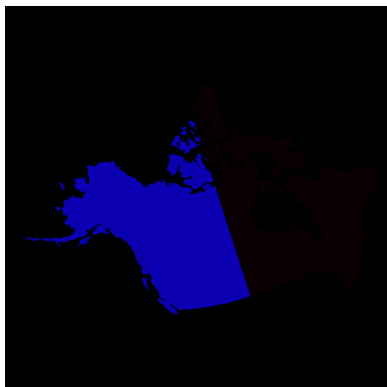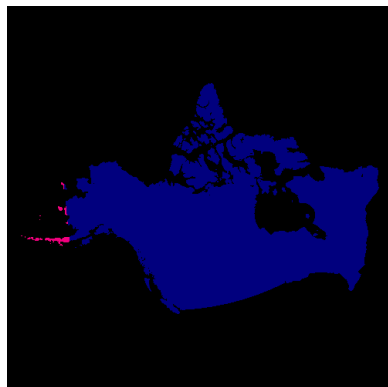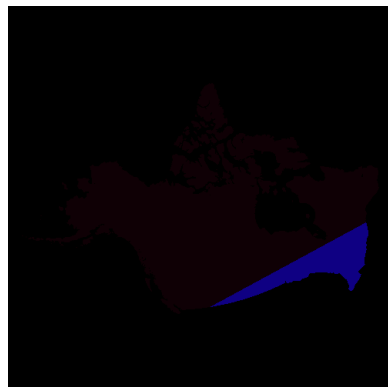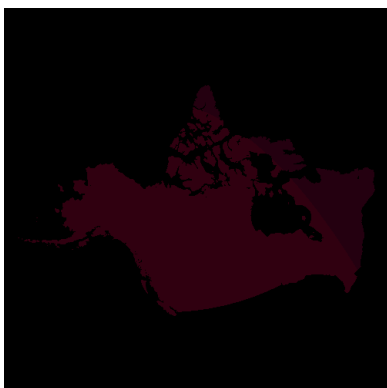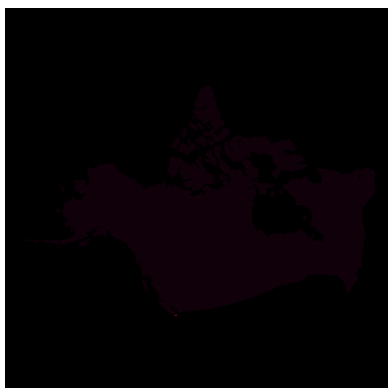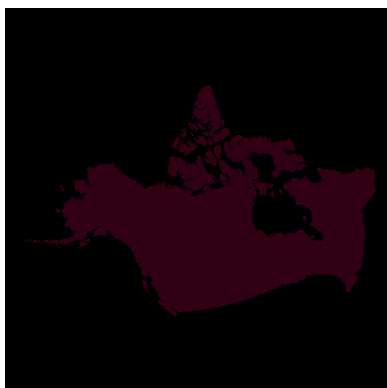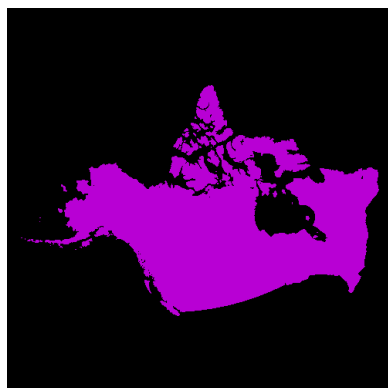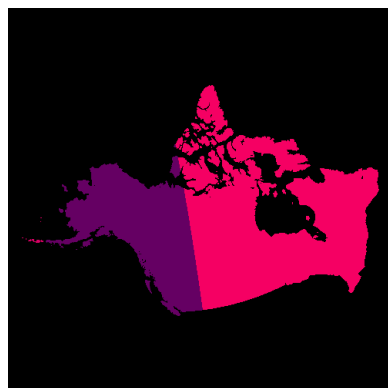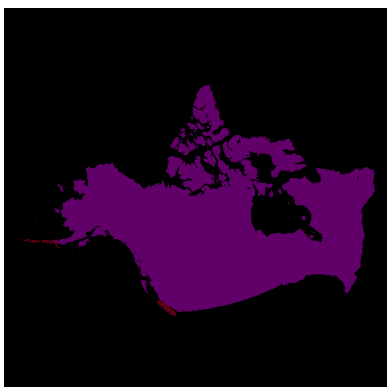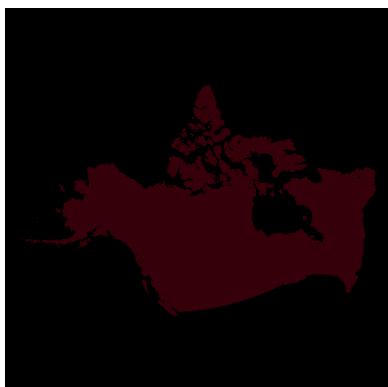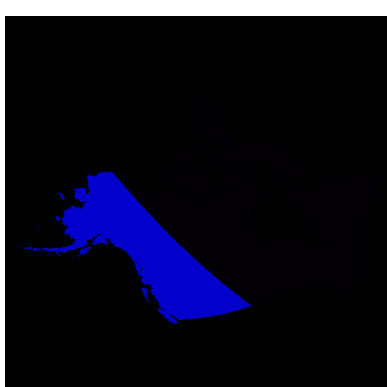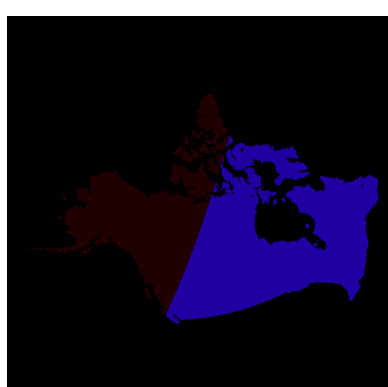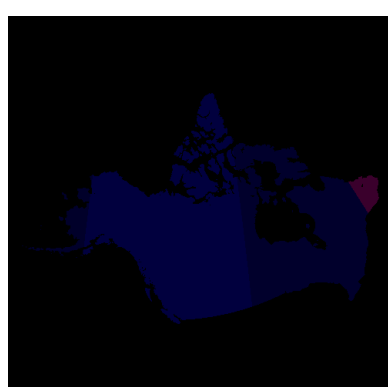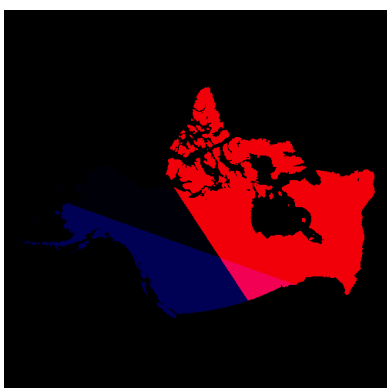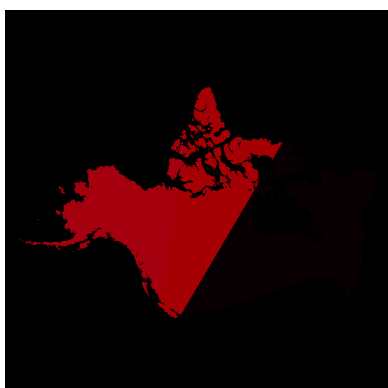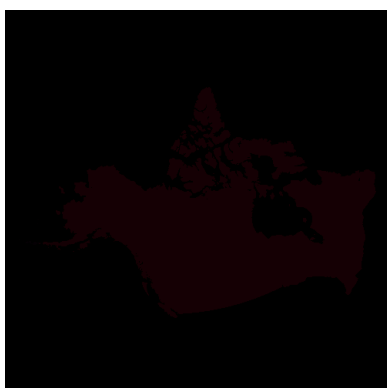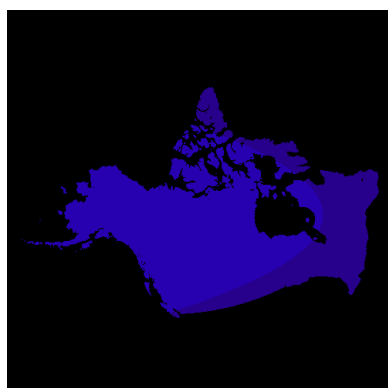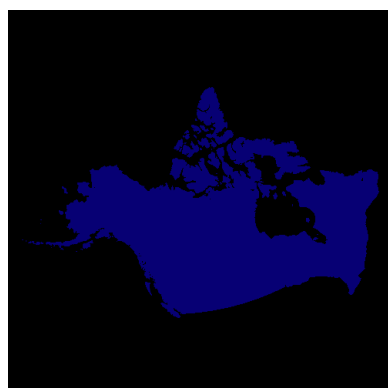

Supplement: supplement [file NIHMS2092588-supplement-supplement.zip › wolf_train.pdf]
